# Spatiotemporal reconstruction of ancient road networks through sequential cost–benefit analysis

**DOI:** 10.1093/pnasnexus/pgac313

**Published:** 2023-01-19

**Authors:** Maximilian J Stahlberg, Guillaume Sagnol, Benjamin Ducke, Max Klimm

**Affiliations:** Institute of Mathematics, Technical University of Berlin, Straße des 17. Juni 136, 10623 Berlin, Germany; Institute of Mathematics, Technical University of Berlin, Straße des 17. Juni 136, 10623 Berlin, Germany; Central Research Services, German Archaeological Institute, Podbielskiallee 69–71, 14195 Berlin, Germany; Institute of Mathematics, Technical University of Berlin, Straße des 17. Juni 136, 10623 Berlin, Germany

**Keywords:** archaeology, spatial networks, path dependence

## Abstract

The construction of ancient road networks spanned generations and exhibits temporal path dependence that is not fully captured by established network formation models that are used to support archaeological reasoning. We introduce an evolutionary model that captures explicitly the sequential nature of road network formation: A central feature is that connections are added successively and according to an optimal cost–benefit trade-off with respect to existing connections. In this model, the network topology emerges rapidly from early decisions, a trait that makes it possible to identify plausible road construction orders in practice. Based on this observation we develop a method to compress the search space of path-dependent optimization problems. We use this method to show that the model’s assumptions on ancient decision-making allow the reconstruction of partially known road networks from the Roman era in good detail and from sparse archaeological evidence. In particular, we identify missing links in the major road network of ancient Sardinia that are in good agreement with expert predictions.

Significance Statement The growth of infrastructure networks is driven and constrained by economic, political, and social phenomena whose impact can be studied through mathematical models. An aspect rarely modeled is how a network is shaped by the historical path of its gradual formation, as early links may form a backbone that affects the course of subsequent connections. We study here a model that considers such path dependence in concert with economic considerations to explain the formation of ancient road networks, and we develop algorithmic tools to allow for historical paths to be estimated. When a possible construction history is inferred from sparse archaeological evidence, the model can make reasonable predictions about links not yet discovered.

## Introduction

Formalized road networks are a fundamental signature of civilization and shape the spatial extents of human interaction. Ranging from prehistoric tracks and the vast network of *viae* that spanned the Roman Empire to our modern highways, a road represents an artificial corridor of reduced movement costs. Its initial construction and continued maintenance both come at an expense that human decision-makers need to find reasonable for the road to come into existence and, respectively, to remain in use. A variety of mathematical models have been proposed to describe (transport) network formation as a result of such a cost–benefit trade-off governing link creation (1–13), yet no claims have been made that these models are able to match the known topology of ancient road networks to a precision that would enable predictions about undiscovered links.

Surprisingly, while both the mobility requirements as well as the resources and construction techniques available to mankind have evolved significantly over the millennia, many modern roads are found to be continuations of ancient ones, having outlasted the civilizations that first built them. Network reconstruction techniques that propose links independently of other links or according to a global optimization goal, while popular in archaeological reasoning, are conceptually unfit to account for temporal path dependence in the network formation process (8,14) and, by extension, to explain the stunning longevity of network links under changing regimes. Historical paths were further shown to influence the growth of modern road networks despite the possibility of central planning (15–20). We propose thus a model that represents explicitly both the economic trade-off as well as the causal dependence between new and existing links. We show that both aspects together, implemented as a sequence of local optimization tasks, are suited to explain the final state of partially known networks from the Roman era.

### Network formation

**Fig. 1.**
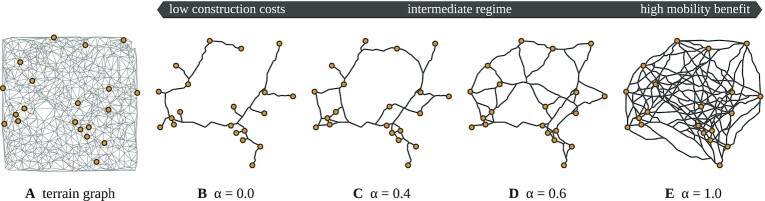
Sequential road networks for different values of α. (A) The artificial terrain graph *G* obtained from a Delaunay triangulation of uniformly distributed points, with randomly chosen sites *S*. Edge costs *c* are proportional to the Euclidean length of an edge (flat terrain). (B)–(E) Road networks obtained using sequential cost–benefit analysis with a fixed connection order and varying trade-off parameter α. Sequential decisions in (B) only minimize construction costs, in (E) only maximize benefit (i.e., low travel cost).

Our model is based on three simplifying assumptions concerning the growth of ancient road networks: First, roads are added in sequence as their construction is fast compared to the lifespan of a network. Second, traveling through the wilderness is prohibitively difficult, so that the road network will eventually contain adequate connections between all pairs of significant places. Last, the course of such connections optimizes a trade-off between road construction and travel costs at the time at which they are established. From these requirements we derive a sequential network formation procedure that we formalize as follows: Let *G* = (*V, E*) be a *terrain graph* containing potential road segments (edges *E*) and junction points (vertices *V*) (see Figs 1A and 4A). Every edge *e* ∈ *E* is equipped with a non-negative cost *c_e_* that encodes the friction imposed by the terrain on both travel and road construction efforts. More precisely, we assume that initial construction costs are (1 − α)*c_e_* and that ongoing travel costs amount to α*c_e_*, where α ∈ [0, 1] is a parameter of the model. Let further *K* be a set of *connections* to be established in *G*, each an unordered (undirected) pair of vertices, and let π be an ordering of *K*. Then, a road network is formed by *establishing* the connections in *K* in order of π: To establish a connection {*u, v*} ∈ *K*, compute a least cost *u*-*v*-path *P* in *G*, add all its nonroad edges *e* ∈ *P*∖*R* to the set of *road segments R* (initially empty), and multiply the cost of edges that were newly added to *R* by α, making them cheaper in subsequent iterations. Like this, construction costs are only paid once while travel costs are experienced by all connections using the road segment. When all connections are established, return the subgraph of *G* comprising all road segments and adjacent vertices as the final road network. We call this a *sequential road network* (SRN).

**Fig. 2.**
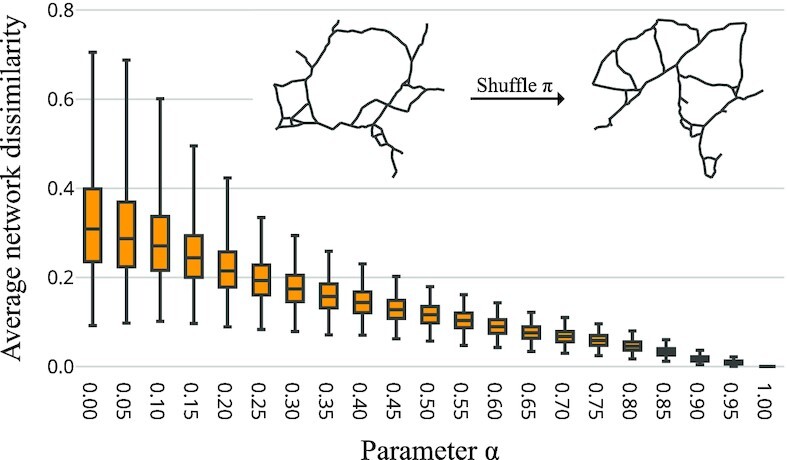
Relative magnitude of path dependence for varying α. *G* and *S* as in Fig. 1. For every value of α, 5,000 pairs of road networks produced from i.i.d. and uniform connection orders are compared (see “Materials and Methods”). A value of 1 denotes the largest dissimilarity observed; whiskers show first and 99th percentile. (Inset) Exemplary network topology effect of resampling the order π for α = 0.4.

**Fig. 3.**
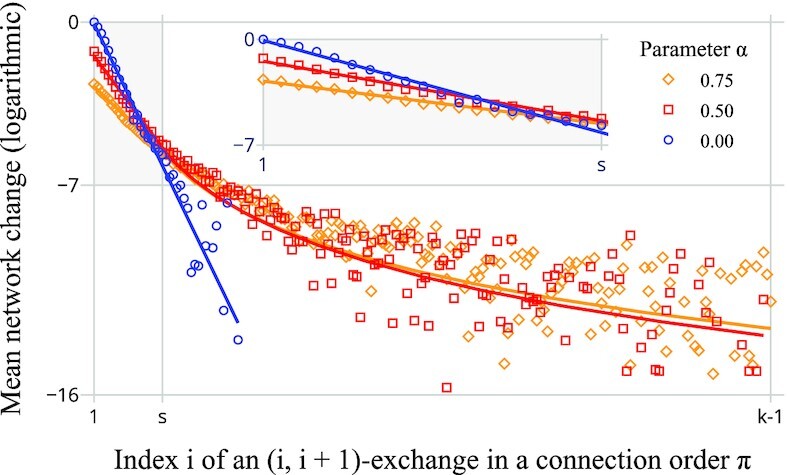
Vanishing impact of atomic changes around later positions *i* in a connection order of length *k*. Terrain and set of *s* sites as in Fig. 1. Impact is measured as the mean dissimilarity δ between a network produced from a random connection order and the network obtained from the same order with the *i*th and (*i* + 1)th connection exchanged; δ = 1 (log δ = 0) denotes the largest mean observed. Every point represents 10,000 comparisons; averages of zero are omitted. We observe empirically a phase of exponential decay for *i* ≲ *s* (inset) and a power–law regime for *i* ≳ *s*. For α = 0, the decay appears exponential throughout. Solid lines show a functional fit that is consistent with these observations.

**Fig. 4.**
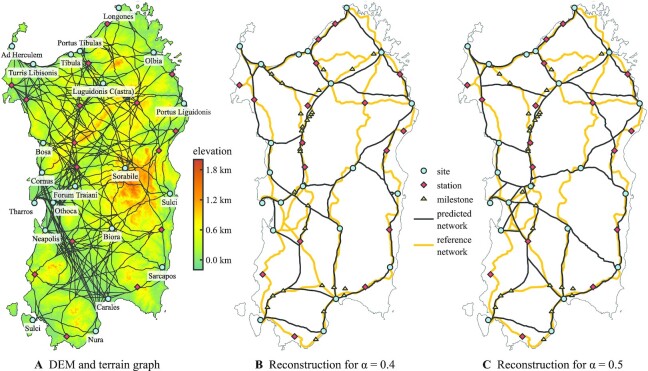
Evidence-based reconstruction of the Roman road network on Sardinia. (A) Terrain graph of potential road segments above a digital elevation model (DEM) of Sardinia. The graph is obtained as the union of least-cost paths between all pairs of places visited by the *Itinerarium Antonini* (IA), where costs depend on the slope induced by the DEM. In addition to the places shown, the graph has nodes at all intersections. (B) and (C) Two road networks produced by our model given the terrain graph, a selection of sites from the IA, and different values of α, with the connection order chosen to minimize the distance to a number of points that provide evidence of a nearby road (stations, milestones).

Matching the assumption of pairwise adequate connectivity, we assume in the following that the connection set *K* contains all pairwise connections between a set of (archaeological) *sites S* ⊆*V*. While this choice implies that all sites share a time frame of concurrent activity, it remains possible to model the formation of new sites by delaying the first connection that involves them within the connection order π.

Maintenance costs may be modeled in a limited fashion by considering them “up front” alongside road construction costs: the future benefits of obtaining a short connection are then weighted against prospective maintenance costs alongside one-time construction fees. For simplicity, we refer only to construction costs in the following.

From the perspective of two sites that aim to form a connection, the parameter α traces a cost–benefit curve: If α = 1, then edge costs remain constant so that new road segments are constructed along the initial least-cost path for every connection. If α = 0, then existing roads are assigned a cost of zero and the procedure minimizes for every connection only the amount of new road segments built, accepting detours along existing roads of any length. Intermediate values interpolate between these extremes and reflect the relative importance of the sustained use cost of network links (i.e., travel time) compared to the one-time cost of building them. For example, when α = 0.5, then a shortcut must cut travel costs in half to be worth the construction effort.

Looking at the network formation process as a whole, one may think of α as a disposition toward infrastructure investments: lower values lead to sparser networks that are cheap to build but impose large travel distances on their users; higher values lead to densely connected networks that are expensive to construct but offer shorter, more direct site-to-site paths. Figure 1B to E shows road networks produced from a fixed connection order and for varying α on artificial flat terrain.

While α conditions the economic dimension of the model, the construction order π captures the historical path of network link formation. The earliest connections in π see few pre-existing road segments and tend to be established along least-cost paths with respect to the initial costs imposed by the terrain. For α < 1, later connections are less likely to follow such “direct” paths but will instead make detours along existing road segments, for which construction costs were already paid. This explicit representation of temporal path dependence leads to a parameter space (possible values for π) that grows extremely quickly with the number of sites in *S* (see section “Computational Complexity”). A central contribution of the present study is an analysis of this complexity and a set of algorithmic tools that make it possible to estimate a plausible construction order π already from sparse data on a network’s final state. Our method makes use of the observation that the earliest connections are by far the most important.

## Results

### Effects of cost reduction

For the extreme case of α = 1, the resulting road network is independent of the chosen connection order π so that effects of path dependence vanish. If, further, *G* is obtained from a digital elevation model (DEM) such that vertices correspond to cells and edge costs are based on the distance and slope between neighboring cells of the DEM, then we obtain as a special case of our model a network prediction based on *least-cost path analysis* (LCPA) (21–24). For flat and uniform terrain, edge costs in this setting reduce to the Euclidean distances between adjacent cells so that connections are established by straight paths and redundant (i.e., close and approximately parallel) path segments are likely to emerge (see Fig. 1E). Elevation-based LCPA is, therefore, best applied to mountainous terrain, where spatially related paths converge in natural movement corridors like valleys and ridges already without cost reduction effects (22), whereas it yields implausibly dense network predictions in flat areas.

For α < 1, movement costs are no longer constant but depend on the current state of the network being formed: road segments built to establish early connections may now serve as cheap movement corridors for later connections. As α decreases, the impact of existing roads on the movement cost metric grows stronger, leading to an increased importance of the historical path that is described by π. This can be quantified by measuring the dissimilarity between road networks generated on the same terrain and with equal sites but with the connection order chosen uniformly at random (Fig. 2).

For the opposite extreme case of α = 0, the resulting network is a Steiner tree (i.e., an acyclic graph connecting the terminals via intermediate nodes), as the path establishing the first connection introduces no cycle and any further path introducing a cycle to the road network would not have minimal cost. Moreover, if instead of the discrete graph *G* the procedure is executed in the Euclidean plane with shortest paths being composed of line segments, then any junction point that is not a site will have only right or straight angles between its incident path segments (25). Thus, up to trivial instances the Steiner tree obtained is suboptimal (otherwise it would have angles of 120° at junctions (26)), as can be expected from a naturally grown network.

While we established that α controls both network density and the magnitude of influence that the connection order π has over the final shape of the road network, it is not yet clear which connections of π exercise this influence. By measuring the effect of a local change at varying positions of a connection order on the resulting network, we find that the network topology emerges rapidly during early stages of network formation, leaving little room for later connections to influence one another. More precisely, the mean network change caused by adjacent transposition (exchange of consecutive elements) at increasing positions of a connection order appears to decay quickly and according to a split regime: For *s* = |*S*| sites, the effect of changes within the first approximately *s* positions can be adequately described by exponential decay, thereafter by a power law. This regime shift is depicted in Fig. 3 (for linear and log–log plots see Fig. S5) and is reminiscent of structural breakpoints in the evolution of random graphs: If we interpret *S* as the vertices of an initially empty graph *H* to which we successively add as edges the elements of *K* in a random order π, then *H* describes an Erdős–Rényi random graph process. Therein, almost surely for *s* → ∞, a large connected component emerges abruptly and grows to compose the whole graph when the number of edges passes *s*/2 and *s*/2 · log *s*, respectively, while *H* contains isolated vertices almost surely before the latter threshold is reached (27). As connected components in *H* imply connected subgraphs in the road network formed by π in *G*, it follows that *s*/2 · log *s* (≈ 1.5*s* in Fig. 3) is also an asymptotic and probabilistic upper bound for the number of connections that need to be established before every pair of sites in *S* is connected by road. In the road formation process, however, connected components can also merge as a result of roads joining, crossing, or passing through formerly isolated vertices, so that we can expect the number of connected components to approach one even more rapidly. This does not finalize the road network, however, as existing routes may still be improved by shortcuts. We suspect that the transition from establishing initial connectivity to building shortcuts corresponds to the observed regime shift in the decay of influence for later connections. In the section “Heuristic solution via Euclidean embedding of permutations,” we exploit this rapid decay of influence algorithmically. We extended the analysis of Fig. 3 to additional graph structures and site distributions in Fig. S4.

### Reconstruction of Roman road networks

We evaluate the reconstruction capabilities of our model on Roman Sardinia, whose road network is to this day subject of a lively archaeological debate (28, 29). As tangible evidence is sparse, scholarly reconstructions of the network rely heavily on the *Itinerarium Antonini* (IA), an ancient text that mentions four north–south routes along the island, each represented by a sequence of places visited and the distances between them in Roman miles. Not all of the places listed can be located with certainty and it has proven hard to embed the routes on the surface of Sardinia such that all distances in the IA are obeyed, in particular in the northern part of the island (29). Unlikely to contain a complete record of all major roads at the time, the document mentions only a single east–west route, a route *per compendium* (shortcut) from *Portus Tibulas* to *Olbia*, with the location of the former site being debated to this day. The interpretation of (28) is shown as a reference network in Fig. 4.

To compare our model’s prediction with existing network reconstructions, we also limit the analysis to places mentioned in the IA, using Fig. 37 of (28) for their suspected locations (excluding *Viniolae* and *Erucium*, which are not charted therein). However, we do not use any route or distance information from the IA. Instead, we propose a reasonable partition of the places mentioned into a group of 21 *sites*, comprising major settlements (*oppida*) and other places that we expect to have motivated the construction of roads, and into 17 *stations*, comprising way stations (*mansiones*), smaller villages (*vici*), and other waypoints of the IA that were possibly constructed as a consequence of a major road already existing (see “Materials and Methods” for details on the partition). To the set of stations we add the 32 retrieval locations of Roman milestones reported in Fig. 37 of (28) as additional indicators of a nearby road, together comprising a set of *evidence* points. We then use a search heuristic with domain-specific preprocessing to find an order of road construction between all pairs of selected sites whose resulting network explains the evidence.

To obtain a graphical representation *G* of the terrain surrounding the sites, we compute from a DEM of Sardinia (30) a slope map with a resolution of 50 m. We then apply to each of its cells Tobler’s hiking function to produce a movement cost surface (raster whose cells represent a cost to traverse) (31, 32). Thereon, we compute for every pair of places mentioned in the IA a least-cost path. The paths converge in natural movement corridors that we treat as candidate road segments, inspired by an analysis in (21). More precisely, we form the union over all paths and introduce additional junction nodes where path segments cross or join. The DEM and resulting terrain graph *G* are shown in Fig. 4A.

The use of a well-known and simple movement cost metric based on freely available, general purpose topographic data—specifically a global-scale grid of terrain elevation produced by NASA’s *Shuttle Radar Topography Mission* in the year 2000 (33)—puts the focus of this case study on reproducibility and on our model’s ability to recover the high level topology of a road network. Thus, one should not expect highly accurate predictions of individual network links as they may have existed in the past. In particular, the spatial resolution of 50 m facilitates the convergence of paths, thereby reducing the number of edges of *G*, but may conceal thin corridors of movement through difficult terrain (34). The hope is that such errors remain insignificant on the scale of the study region (∼10^7^ grid cells) by leaving the general course and cost of most paths intact. Additionally, one should keep in mind that LCPA proposes idealized corridors, which do not need to correspond with historical roads (34) even at a higher resolution or when additional layers of data besides elevation (35) are processed by more elaborate cost functions (36).

As an alternative to the graphical representation *G*, one could also execute the network formation procedure directly on a raster cost surface, reducing the cost of its cells in place of edges of *G*. This approach is, however, impractical for testing a large number of connection orders unless the resolution of the cost surface is reduced further.

**Fig. 5.**
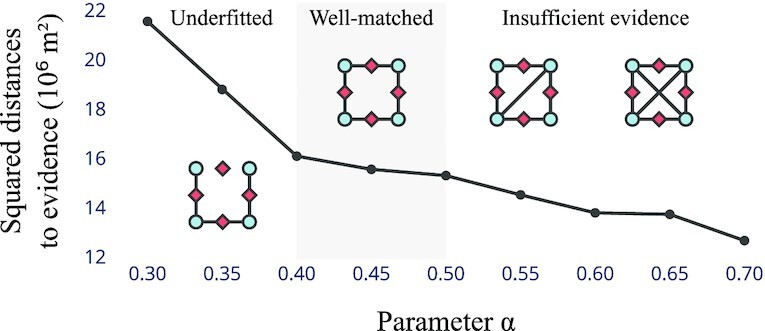
Sum of squared distances attained between the reconstructed network and the points of evidence to which it is fitted, for varying α. The sharp decrease for α < 0.4 indicates that the road network is too sparse to explain all evidence (underfitting). On the other hand, the sparsity of the evidence causes overfitting at higher α; we infer from visual inspection of the results that this happens around α > 0.5.

Finally, we perform evidence-based reconstruction for α ∈ [0.3, 0.7] with a step size of 0.05. That is, we fix α and search for an order of connections such that the resulting road network minimizes the sum of squared distances between the evidence locations and the network; see section “Heuristic solution via Euclidean embedding of permutations” for the procedure. We find that the evidence is well-matched but is too sparse to explain almost all emerging road segments for α > 0.5 and that, on the other hand, the road network becomes too sparse to cover the available evidence for α < 0.4 (Fig. 5). In contrast, the network predictions for α ∈ [0.4, 0.5] cover the evidence well and extrapolate from it carefully. The cases of α = 0.4 and α = 0.5 are presented in Fig. 4B and C, the remainder in Fig. S6. When we compare the two results selected with the network charted in Fig. 37 of (28), which is based on expert knowledge and was not made available to the search procedure, we find that both predictions feature a surprising number of road segments that match this reference closely. In particular, we can recognize three of the four north–south routes of the IA within both reconstructed networks, apart from a missing stretch of road between *Neapolis* and southern *Sulci*. From the reconstructed networks, we further obtain plausible hypotheses on the location of east–west routes that are not represented in the IA: First, both predictions propose a branch connecting *Bosa* to the stretch of road between *Forum Traiani* and *Luguidonis C(astra)*, which is also found in the reconstruction of (41). Second, both predictions show *Sorabile* as a potential hub for travel between the western inland sprawl and the eastern coast. A connection between *Forum Traiani* and *Sorabile* can also be found in the reconstructions proposed in (41) and (42), while (28) argues that a road between *Sorabile* and the coast could have existed. That our model predicts these segments is remarkable as they are not covered by the sparse evidence to which it was fitted. Instead, they emerge as a consequence of a connection order that was identified to explain evidence elsewhere.

**Fig. 6.**
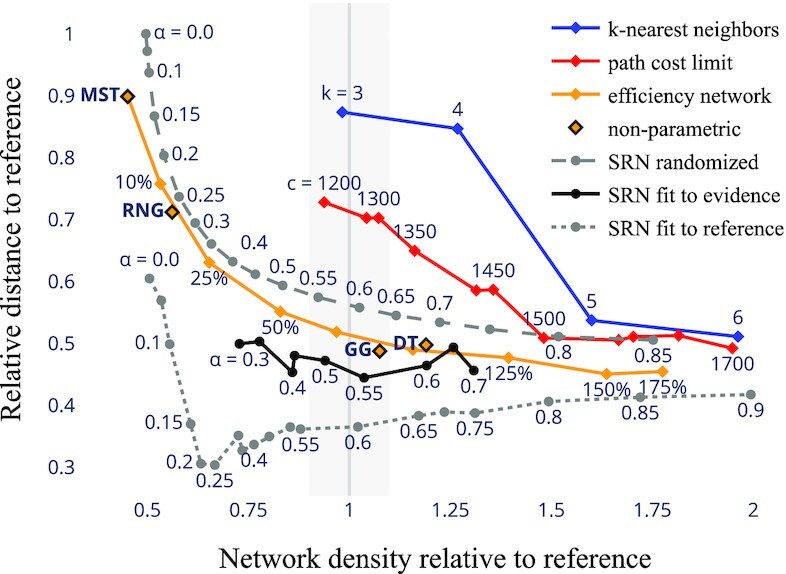
Comparison of reconstruction results with established models. All models are executed on the same elevation-based terrain graph defining site-to-site travel costs (Fig. 4A). Horizontal axis: network density (sum of road segment lengths) in relation to the reference network (Fig. 4); the gray stripe marks a $10\%$-region around the reference density. Vertical axis: dissimilarity between model outcome and reference network (see “Materials and Methods”), relative to the largest value observed. We expect a good reconstruction to have relative density around 1 and low dissimilarity. (Blue) Sites are connected to their *k* cost-nearest neighbors. (Red) Connections to neighbors at most *c* cost-units away. (Orange) Efficiency networks (12) as stated in (37). (Diamonds) Minimum-cost spanning tree (MST), relative neighborhood graph (RNG) (38), Gabriel graph (GG) (39), and Delaunay triangulation (DT) (40). (Dashed gray) SRN using random connection orders (average of 1,000 networks). (Black) SRN fit to sparse evidence (section “Reconstruction of Roman road networks”). (Dotted gray) SRN fit directly to the reference network, showing a best-case estimate of model capabilities in the presence of dense evidence (average of 5). Selected networks are visualized in Figs S6–S11.

We further compare our results quantitatively with seven established models for spatial network recovery (12, 24, 37–40); see Fig. 6. All models are executed on the terrain graph (Fig. 4A), using least-cost paths to determine site distances where appropriate. For the previously identified relevant range of α ∈ [0.4, 0.5], our model (black line) produces networks that have a similar density as the reference network and lower dissimilarity to it than the other models. This is achieved by learning a construction order from a sparse set of spatial reference points but without using the reference network as an input. The other models are not designed to be informed by spatial evidence beyond the sites being connected, so we note that they are at an information disadvantage. However, we stress the possibility to learn from “roadside evidence” such as milestone locations as a feature of our model, as such data is often available in practice. To test out the model’s limits as the amount of evidence varies, we perform two more artificial tests: First, we measure the average dissimilarity attained when the order of connections is chosen at random, thus using no more data than the competing models (gray dashed line). Second, we make the complete reference network available to the search procedure (gray dotted line). In the first case, the performance is undistinguished with respect to the competing models. This highlights the importance of historical paths in our model but also shows that the space of networks that can be generated by it is not too large, as the remaining (economic) assumptions underlying the model are not entirely dominated by the construction order. In the second case the result is greatly improved, indicating that the predictive capabilities of our model are, on the other hand, not exhausted by the sparse evidence that we have provided it with. Together these bounds outline a moderate expressiveness of the parameter π, which could render our model more robust to overfitting than the vast range of possible values for π would suggest. However, we note that the reference network itself is an estimate that is informed substantially by the same set of evidence, so that its value in measuring a generalization error is limited.^1^

Apart from predictive quality, information requirements and computational costs are deciding factors in model selection. This remains a strength of the competing models, which do not require a data-driven parameterization and are, therefore, quick to implement and cheap to compute.

### Computational complexity

One may wonder if it is possible to find an optimal connection order algorithmically or whether our use of a heuristic search strategy is justified. We argue the latter via a formal proof of computational hardness. Note that there are $\binom{s}{2}!$ possible connection orders for *s* = |*S*| sites, so it is intractable to simply evaluate all of them already for *s* > 5. Two problem variants are natural candidates for analysis: First, we have shown practical interest in an order whose resulting network coincides with prior knowledge. For rigorous study we represent this task by a simplified decision problem: Given a reference network defined as a subgraph of the terrain graph, decide whether there is an order such that the resulting network differs from the reference in at most *k* edges. Second, we might be interested in an order that minimizes the total cost of establishing paths chosen by the network formation procedure: The relationship between such a best-case sequential network and one that is designed by a central authority yields a lower bound on the inefficiency of naturally grown road networks.^2^ We prove that the first problem variant is computationally intractable (NP-hard) to solve exactly for $\alpha \in [\frac{1}{2}, 1)$ and that the latter variant is intractable for α < 1 and, under standard assumptions, also admits no efficient algorithm to approximate the exact solution to arbitrary precision (the problem admits no PTAS unless P = NP). Despite the latter result, there is a lower bound for the quality achievable by a constant-factor approximation that is related to the statistical findings in Fig. 2: For α > 0, any connection order is an $\frac{1}{\alpha }$-approximation of the cheapest one. The theorems are formalized and proven in Proposition 1, Theorems 1 and 2, and Corollary 2 (Supplementary Material; proof ideas shown in Figs S1–S3).

### Heuristic solution via Euclidean embedding of permutations

A vast number of methods have been proposed for the heuristic solution of hard optimization problems over the space of permutations, in particular to tackle the Traveling Salesperson Problem (45, 46). A key assumption underlying these methods is that similar inputs lead to similar outputs, so that better solutions can be expected in the vicinity of good solutions. This assumption, however, does not hold for path-dependent processes: The natural candidate for a minimal change in a permutation is adjacent transposition but we have shown that for a connection order, the impact of such a change depends critically on the positions of the connections being exchanged. To adapt established solution techniques to this asymmetric setting, we present an embedding of the *solution space* of connection orders into a high-dimensional Euclidean *search space* such that close points in the search space correspond to similar networks on average.

**Fig. 7.**
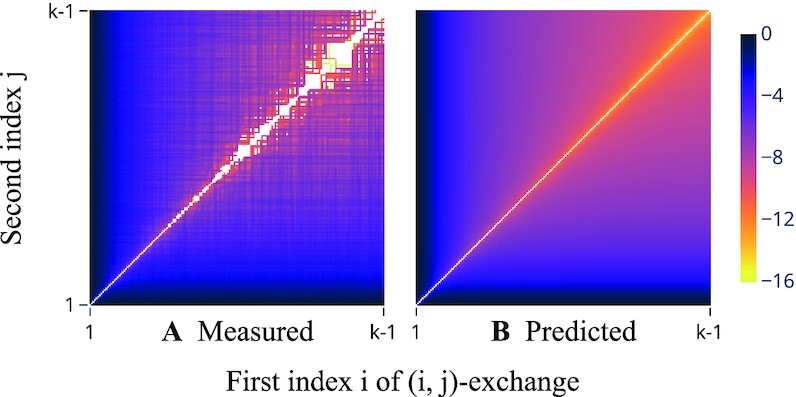
Logarithmic plot of the average network change when swapping positions *i* and *j* in a random connection order. Setup as for Fig. 3 with α = 0.5. A log-value of 0 (black) denotes the largest average observed. Averages of zero (no change observed) are shown in white. (A) The measured dissimilarity matrix *D*; each entry represents the average of 1,000 comparisons. (B) A smooth prediction of *D* computed only from the functional fit *f* for α = 0.5 (red line) of Fig. 3: we set the diagonal to zero and, for *i* < *j*, entries (*i, j*) and (*j, i*) both to $\sum _{\ell = i}^{j - 1} f(\ell )$.

We have already quantified the effect of adjacent transposition at varying positions of a connection order (Fig. 3). As we extend this analysis to the effect of nonadjacent transposition (Fig. 7), we uncover that a sharp triangle inequality holds: When *D_ij_* denotes the average dissimilarity between two networks whose connection orders are equivalent up to an exchange of positions *i* and *j*, then *D_ij_* ≈ *D*_*i*ℓ_ + *D*_ℓ*j*_ holds for all *i* < ℓ < *j*. This property will be useful as it allows us to construct the Euclidean embedding based only on (computationally cheap) statistics on the effect of adjacent transposition but in such a way that distances in the search space approximate also the effect caused by larger changes.

The embedding is obtained as follows. First, we define a compositional (positive, summing to one) weight vector *w* based only on the superdiagonal *d* of the matrix (*D_ij_*), which contains measurements on the effect of adjacent transposition at various positions (*d_i_* corresponds to an exchange of positions *i* and *i* + 1). More precisely, we fit a decreasing function *f* to *d* using prior knowledge about the two regimes of decay that we have identified (section “Effects of cost reduction”), and we use this function to define $w = \Vert w^{\prime } \Vert _1^{-1} w^{\prime }$ where

(1) \begin{eqnarray*}
w^{\prime }_i = \left\lbrace \begin{array}{@{}l@{\quad }l@{}}1, & \text{if}\,i = 1, \\
w^{\prime }_{i - 1} e^{\frac{f(i - 1)}{\sqrt{2}}}, &\text{if}\,1 \lt i \le |K|. \end{array}\right.
\end{eqnarray*}

If π is a connection order and *P*_π_ its associated permutation matrix, then the function Ψ(π) = *P*_π_*w* maps π to a unique point on a high-dimensional unit simplex. We show that for a metric on simplices called the *Aitchison distance* (47), *d_A_*, we have *d_A_*(Ψ(π), Ψ(π^*ij*^)) ≈ *D_ij_*, where π^*ij*^ is equal to π with the *i*th and *j*th positions exchanged. We may then use the fact that the Aitchison geometry is isometric to regular Euclidean space as witnessed by the *isometric log-ratio transformation* (48), ${ilr}$, to obtain the embedding $\Phi (\pi ) = {ilr}(\Psi _w(\pi ))$ with the desired property that ‖Φ(π) − Φ(π^*ij*^)‖ ≈ *D_ij_*. A detailed version of this construction with the omitted proofs and a conic quadratic program to compute *f* are found in the Supplementary Material.

**Fig. 8.**
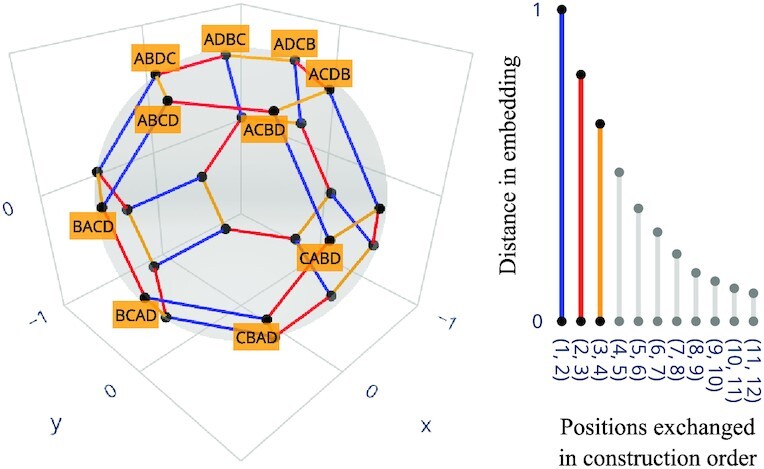
Embedding of all orderings of the first four entries of a connection order (represented by the letters A–D) in 3D Euclidean space. Edges represent adjacent transposition at varying positions; their length is by construction proportional to the average network change measured in Fig. 3 for α = 0.5. The transformation Φ extends this embedding such that every connection order is mapped to a point in $\mathbb {R}^{k-1}$, with close points producing similar networks on average.

In Fig. 8, the image of the embedding Φ is visualized for the low-dimensional case of *k* = 4 connections. It can be seen that all permutations are embedded onto a (*k* − 2)-sphere (here a 2-sphere embedded in $\mathbb {R}^3$), whose radius is approximately $\left \| {D} \right \| _{\text{F}}/(2 {\sqrt{k}})$ (Supplementary Material). In higher dimensions, we have by construction that points corresponding to connection orders that differ only in late positions are collapsed into regions whose sizes correspond to the negligible expected difference in the associated road networks. One may thus think of the search space as a compressed version of the solution space. This preprocessing makes the computationally hard search for good connection orders amenable to established derivative-free optimization methods, as demonstrated by our reconstruction results.

## Discussion

We propose and study a mathematical model to describe the evolution of ancient road networks based on a number of reasonable assumptions, the most fundamental being a substantial dependence of the course of new roads on the existing network structure, which was not unravelled algorithmically in earlier work. Through this, we tackle the archaeological challenge of understanding the long term effects of short-term human decision-making (49). The explicit representation of temporal path dependence poses computational challenges that we approach with a data-driven transformation of the emerging search space. This makes the computation of explicatory connection orders feasible and allows us to produce networks that are in good agreement with scholarly predictions of ancient road networks.

Alongside path dependence, we consider as a driving factor of network evolution a cost–benefit trade-off that controls where, if any, new road segments are built when a connection is scheduled to be formed. We find that this process produces reasonable network reconstructions, on average, even when the connection sequence is randomized. Thus, economic and historical conditions act on the network configuration in a complementary manner. Other driving factors are not modeled explicitly and can, at best, be encoded in the connection sequence or the terrain graph. For instance, a road could form as a result of political calculation or military necessity, or be impeded by administrative discordance or safety considerations. In the case study presented, such considerations could be significant for the *Barbaria*, a region spanning Sardinia’s eastern inland that has resisted Roman rule substantially (28).

While our method yields a road construction history that explains the predicted network structure, the scarcity of ancient roads whose initial construction time is precisely known stands in the way of an empirical evaluation of such temporal predictions. This raises, however, an interesting theoretical question for future research: To what extent can similar networks emerge from dissimilar histories? Our analysis of the relative importance of positions in a connection order may be seen as a first step toward an answer.

In the quantitative comparison, we outperformed established models by learning a construction order from an additional body of spatial evidence. It would certainly be interesting to develop and study extensions of the tested and of further models, which can also be informed by such (possibly noisy) data. For instance for gravity models (9,24), which we exclude from our comparison as the size of settlements in the study region is not well-known, it could be feasible to estimate that size from incomplete knowledge about the road network. An independent review of such informed models, applying the testing methodology and possibly some of the algorithmic tools that we introduce, might be of interest to practitioners.

Further, one may consider the model proposed as a baseline model for more complex network dynamics. In particular, while we assume here uninterrupted growth into a final network state, it would also be possible to consider *disconnection* events within the construction sequence π, which lead to the abandonment of unused links. This way, the network can be shaped in a form not entirely defined by the settlements that remain eventually. Such a model may seem more appropriate for diachronic studies spanning time frames of network decay but it is questionable whether enough data exists to validate the added complexity. Likewise, it may be useful to model link maintenance costs separately from setup costs, such that these costs may increase retroactively and render existing road segments too costly to maintain.

Beyond archaeology, artificial transport networks enable the study of failure robustness, congestion effects, or algorithm performance under varying network size and density assumptions (50,51). As the parameter α interpolates smoothly between extremely sparse and extremely dense graphs while maintaining a set of reasonable assumptions on the structure of gradually constructed road networks, networks produced by our model can be used to this end. In particular, the environmental conditions met by contemporary networks can be encoded in the terrain graph, which does not need to be planar.

Finally, the proposed strategy of distributing permutations over a high-dimensional hypersphere collapses large low-variance regions in the solution space to point clusters of negligible size in the search space. This approach is not limited to the application discussed here but can be applied more generally to hard optimization problems over the space of permutations with the property that early decisions have a greater importance than later ones. An example are time-dependent variants of the Traveling Salesperson Problem with applications in scheduling (52–54).

## Methods

### Interactive visualization tool

An online tool to visualize SRNs and their development over time is presented at https://wsgi.math.tu-berlin.de/roadnets/.

### Artificial terrain generation

To obtain the terrain graph shown in Fig. 1A, we distribute 900 points uniformly on [0, 1]^2^ and compute a Delaunay triangulation thereof to produce a spatial graph structure with ca. 2,700 edges. Edge costs are set to the Euclidean distance and 20 designated sites are selected from all vertices uniformly and without replacement. Figure 1 may be reproduced with the online visualization tool by setting the terrain and road random seeds to 1 and by moving the α-slider (default values elsewhere).

### Network dissimilarity measure

To compare networks with common designated sites, we compute all site-to-site least-cost paths in both networks, then sum over every site pair the area enclosed between both corresponding paths. In the setting of Fig. 1, path segments are straight lines. For Sardinia, we simplify the detailed path segments visible in Fig. 4A using the Ramer–Douglas–Peucker algorithm (55,56) with a tolerance of 500 m.

### Partition of the IA

We select as *sites* 11 places identified with certainty as “oppidum averte ‘rem publicam’ ” (public city) in (57) (p. 605f), five places labeled “città” or “civitas” in (28) (p. 206), the harbors *Portus Tibulas* and *Portus Luguidonis*, the associated *Luguidonis C(astra)*, a major military camp (p. 371), *Sarcapos*, a minor trade center (p. 47), and *Ad Herculem*, which receives mention as a way station (p. 170) but whose location in the extreme northwest of the island (according to the more recent (28, 29), cf. (41, 42)) suggests that a road must have been constructed with *Ad Herculem* or a nearby uncharted place as its destination.

### Search heuristic

We employ a custom EDA to find good (locally optimal) connection orders that exploits the hyperspherical arrangement of the Euclidean embedding and is equipped to deal with multimodal objective functions through adaptive clustering. First, the impact of adjacent transposition (Fig. 3) is measured for the target terrain graph and choice of α to produce the weight vector *w*. A random initial generation of 50(*k* − 1) orders is produced and for each the associated road network is constructed and evaluated according to the objective function at hand. The best $30\%$ of networks are selected, their orders are transformed through Φ, and the resulting points are partitioned into ℓ clusters (initially ℓ = 1) using spherical *k*-means (58). Then, a mixture of von Mises–Fisher distributions (59) is produced by estimating one data-generating distribution for each cluster (60) and setting its weight proportional to the cluster’s size. The next population is sampled from this mixture and the current cluster centroids are used to initialize the next round of *k*-means. If the average solution value of a generation does not represent an improvement over the last, then ℓ is incremented and two instead of one starting centroids are obtained from the cluster with the lowest concentration by sampling from its associated distribution. The above is repeated until 50 generations have been evaluated and the best network found is returned.

### Data processing

Georeferenced data is processed with GRASS GIS (61). The DEM was obtained from the AWMC (30) and is based on data from the NASA SRTM (33). We reproject it from geographic coordinates with a WGS84 datum to planarized coordinates with an ETRS89 datum, and a uniform resolution of 50 m. From this a slope map in degrees is computed (open waters from (30) represented by 30°) and turned into a cost surface with the r.mapcalc command 0.6 * 2.7182818 ^ (3.5 * abs(tan(slope) + 0.05)) (c.f. (31)). Locations of places visited by the IA, milestone retrieval sites, and the reference network shown in Fig. 4 were digitized and georeferenced from Fig. 37 of (28) using QGIS (62).

GRASS GIS plugins to produce terrain and reference graphs, tools to export georeferenced data to Python types, algorithms and analysis tools used throughout the paper, data generation and drawing routines to reproduce all figures, and source code for the interactive visualization tool are all made available under an open source license at https://gitlab.com/ef5-6.

## Supplementary Material

pgac313_Supplemental_File

## Data Availability

Processed data required to reproduce the results are included with the code at https://gitlab.com/ef5-6.

## Footnotes

1 Vice versa, some way stations are not identified with certainty and are located based on beliefs about the road network (29).

2 This quantity is studied in (43) under the name *myopic sequential price of stability* and in (44) as the competitive ratio of a greedy algorithm for the *Online Steiner Forest* problem.
