# Supplementary material for: Spatiotemporal reconstruction of ancient road networks through sequential cost–benefit analysis
Source: PNAS Nexus. 2023 Jan 19;2(2):pgac313. doi: 10.1093/pnasnexus/pgac313 (PMC9944230; doi:10.1093/pnasnexus/pgac313)
Supplement: pgac313_Supplemental_File [file pgac313_supplemental_file.pdf]

PNAS nexus

Supplementary Information for

Spatiotemporal reconstruction of ancient road networks through sequential cost–benefit analysis

Maximilian J. Stahlberg, Guillaume Sagnol, Benjamin Ducke, Max Klimm

Maximilian J. Stahlberg
Email: maximilian.stahlberg@tu-berlin.de

Max Klimm
Email: klimm@tu-berlin.de

This PDF file includes:

Supplementary text
Figs. S1 to S11
References for SI reference citations

Supplementary Information Text

Mathematical preliminaries.

Notation 1 (Integer range)**.** For $n \in \mathbb{Z}_{\geq 1}$ it is $[n] \coloneqq \{k \in \mathbb{Z}_{\geq 1} \mid k \leq n\}$.

Notation 2 (Pairs)**.** For a set X we write $\mathcal{P}_2(X)$ short for $\{Y \subseteq X \mid |Y| = 2\}$.

Convention 1 (Graphs)**.** Graphs are undirected and simple (no parallel edges or loops).

Notation 3 (Neighborhood)**.** For a graph (V, E) and a vertex $u \in V$ we write $N(u) \coloneqq \{v \in V \mid \{u, v\} \in E\}$ for the *(open) neighborhood* of u.

Notation 4 (Path)**.** We formally denote a path in a graph (V, E) by an edge set $P \subseteq E$. We additionally adopt a vertex-based notation and write $P = \{\{v_1, v_2\}, \{v_2, v_3\}, \dots, \{v_{n-1}, v_n\}\}$ as the vertex sequence $(v_1, \dots, v_n)$ or $(v_n, \dots, v_1)$.

Notation 5 (Network)**.** A network (V, E, c) comprises a graph (V, E) and an edge cost function $c\colon E \to \mathbb{R}_{\geq 0}$.

Definition 1 (Progressive road network)**.** Let $N = (V, E, c)$ be a network and let $\pi \in S(K)$ for a set of *connections* $K \subseteq \mathcal{P}_2(V)$ represent a *connection order*. Let further $r(\beta, \tau) : \mathbb{R}_{\geq 0} \times \mathbb{Z}_{\geq 0} \to \mathbb{R}_{\geq 0}$ be a *reduced cost function* that is nondecreasing in a *base cost* β and nonincreasing in a *road tier* τ. Then, the *progressive road network* $R_{N,r}(\pi) \coloneqq (\bigcup F, F, e \mapsto r(c(e), \tau_e))$ with *social cost* $C_{N,r}(\pi) \coloneqq \gamma$ is given by $F \subseteq E$, $\gamma \in \mathbb{R}_{\geq 0}$, and $\tau_e \in \mathbb{Z}_{\geq 0}$ for all $e \in E$ as obtained from the following procedure:

1. Initialize $F \coloneqq \emptyset$, $\gamma \coloneqq 0$, and $\tau_e \coloneqq 0$ for all $e \in E$.
2. For i from 1 to $|K|$:
   (a) Let $\{u, v\} \coloneqq \pi_i$.
   (b) Find a shortest u-v-path $P \subseteq E$ for edge lengths $e \mapsto r(c(e), \tau_e)$.
   (c) Update $F \coloneqq F \cup P$ and $\gamma \coloneqq \gamma + \sum_{e \in P} r(c(e), \tau_e)$.
   (d) For every $e \in P$, increment τ_e.

We assume throughout the document that the shortest path in step 2.b is unique; in practice this can be achieved almost surely by adding random noise of negligible magnitude to the base cost of every edge. It is further assumed that u and v in 2.a are in the same connected component. When N and r are clear from the context, we write $R(\pi)$ for $R_{N,r}(\pi)$ and $C(\pi)$ for $C_{N,r}(\pi)$.

Notation 6 (Establishing paths)**.** When arguing about the procedure in Definition 1, we say that the path P in step 2.b *has (a) reduced cost (of)* $\sum_{e \in P} r(c(e), \tau_e)$ and *establishes* the connection $\{u, v\}$ *at time* i. For $K' \subseteq K$, we say further that a set of paths Q *establishes* K' if every $P \in Q$ establishes a connection $\pi_i \in K'$ at some time i. The paths establishing K are referred to as just the *(π-)establishing paths*.

Definition 2 (Binary cost reduction)**.** The *binary reduced cost function* for $\alpha \in [0, 1]$ is defined as

$$r_\alpha(\beta, \tau) \coloneqq \begin{cases} \alpha\beta, & \text{if } \tau \geq 1, \\ \beta, & \text{if } \tau = 0. \end{cases}$$

Notation 7 (Sequential road network)**.** We call a progressive road network for $r = r_\alpha$ a *sequential road network*.

Notation 8 (Cheap and costly edges)**.** When arguing about the procedure in Definition 1 and $r = r_\alpha$, we say that an edge $e \in E$ is *i-cheap* if $\tau_e > 0$ at the start of the i-th iteration of step 2 (counted from one). Otherwise, we say that e is *i-costly.*

Definition 3 (Sequential road network problems)**.** We define the following family of problems concerning sequential road networks:

Input: A network $N = (V, E, c)$ with $G \coloneqq (V, E)$, a set of connections $K \subseteq \mathcal{P}_2(V)$, and a cost reduction parameter $\alpha \in [0, 1]$. Where applicable, additionally a target cost γ, an edge set $T \subseteq E$, and a polynomial-time computable metric $d\colon \{G[F] \mid F \subseteq E\}^2 \to \mathbb{R}_{\geq 0}$.

Problem: `SRN`
Question: Is there a permutation $\pi \in S(K)$ such that $C(\pi) \leq \gamma$?

Problem: `MIN-SRN`
Output: A permutation $\pi^* \coloneqq \arg\min_{\pi \in S(K)} C(\pi)$.

Problem: `SRN-FIT`
Question: Is there a permutation $\pi \in S(K)$ such that $d(R(\pi), G[T]) \leq \gamma$?

Definition 4 (Weighted symmetric edge difference)**.** Given a graph $G \coloneqq (V, E)$ with edge weights $w\colon E \to \mathbb{R}_{\geq 0}$, a simple metric on edge-induced subgraphs of G is given by

$$d_w\left((V_1, E_1), (V_2, E_2)\right) \coloneqq \sum_{e \in (E_1 \setminus E_2) \cup (E_2 \setminus E_1)} w(e).$$

Definition 5 (Independent and dominating sets)**.** Let (V, E) be a graph. We call a set $U \subseteq V$ an *independent set*, if $\forall u, v \in U\colon \{u, v\} \notin E$, and a *dominating set*, if $\forall v \in V \setminus U\ \exists u \in U\colon \{u, v\} \in E$.

Definition 6 (Independent dominating set problems)**.** For use in reductions we define the following family of hard problems:

Input: A graph $G = (V, E)$. If applicable an integer $k \in \mathbb{Z}_{\geq 0}$.

Problem: `IDS`
Question: Is there a set $U \subseteq V$ with $|U| \leq k$ that is both an independent set and a dominating set in G?

Problem: `MIN-IDS`
Output: A subset $U \subseteq V$ of minimum cardinality that is both an independent set and a dominating set in G.

Fact 1. *`IDS` is NP-complete even on cubic planar graphs. (1)*

Fact 2. *`MIN-IDS` is NP-hard to approximate within a factor of $\frac{681}{680}$ in graphs of degree at most three. (2)*

Definition 7 (Permutation CSP)**.** Another hard problem we use in reductions is closely related to the well known Feedback Arc Set problem:

Problem: Arity 2 Permutation CSP
Input: A set U, a set of constraints $C \subseteq U^2$, and an integer $k \in \mathbb{Z}_{\geq 0}$.
Question: Is there a permutation $\pi \in S(U)$ fulfilling at least k constraints, formally $|\{(u, v) \in C \mid u \prec_\pi v\}| \geq k$?

Fact 3. Arity 2 Permutation CSP *is NP-complete. (3)*

Computational complexity.

Proposition 1. *Any solution candidate $\pi \in S(K)$ to `MIN-SRN` with $\alpha > 0$ is an $\frac{1}{\alpha}$-approximation.*

Proof. Let $N = (V, E, c)$ be a network, $K \subseteq \mathcal{P}_2(V)$ a set of connections and $\alpha \in (0, 1]$ a cost reduction parameter. Let further $\pi^* \coloneqq \arg\min_{\pi \in S(K)} C_{N, r_\alpha}(\pi)$ be an optimum solution to `MIN-SRN` with $R^* \coloneqq R_{N, r_\alpha}(\pi^*)$ the associated road network and $C^* \coloneqq C_{N, r_\alpha}(\pi^*)$ its social cost. We show that then $C^\alpha \coloneqq C_{N, r_\alpha}(\pi) \leq \frac{1}{\alpha} C^*$ holds for every $\pi \in S(K)$. To this end let $\pi \in S(K)$ and consider the network $R^1 \coloneqq R_{N, r_1}(\pi)$ with social cost $C^1 \coloneqq C_{N, r_1}(\pi)$. Since $r_1(\beta, \tau) = \beta$ for every $\tau \in \mathbb{Z}_{\geq 0}$, we have that the π-establishing paths in the formation of R^1 are least cost paths in N. Specifically, the path establishing some $\{u, v\} \in K$ in the formation of R^1 has a reduced cost of $\operatorname{dist}_c(u, v)$. Since further $r_\alpha(\beta, \tau) \geq \alpha\beta$ for every $\tau \in \mathbb{Z}_{\geq 0}$, we have that the path establishing $\{u, v\}$ in the formation of R^* has reduced cost at least $\alpha \operatorname{dist}_c(u, v)$. It is thus $\alpha C^1 \leq C^*$ and it only remains to show that $C^\alpha \leq C^1$. Assume towards a contradiction that $C^\alpha > C^1$. Then, there is a connection $\{u, v\} \in K$ that is established in the formation of C^α at a reduced cost of more than $\operatorname{dist}_c(u, v)$. This however contradicts the fact that a least cost u-v-path P in N has a reduced cost of at most

$$\sum_{e \in P} \max_{\tau \in \mathbb{Z}_{\geq 0}} r_\alpha(c(e), \tau) = \sum_{e \in P} c(e) = \operatorname{dist}_c(u, v). \qquad \square$$

Theorem 1. *`SRN` with $\alpha < 1$ is NP-complete even for $c(E) \subseteq \mathbb{Z}_{\geq 1}$.*

Proof. Containment in NP follows from the fact that the procedure in Definition 1 can be executed in polynomial time and computes $C(\pi)$ for a given $\pi \in S(K)$.

To show NP-hardness, we present a polynomial-time reduction from `IDS`. Let a graph $G = (V, E)$ with $V = [n]$ for some $n \in \mathbb{Z}_{\geq 0}$ and an integer $k \in \mathbb{Z}_{\geq 0}$ compose an instance of `IDS`. We construct an instance of `SRN` given by a network $N = (V', E', c)$ and a set of connections K as follows (see Figure S1 for an example). First, define the vertex set $V' \coloneqq \cup_{i \in [n]} V_i$ where $V_i \coloneqq \{s_i, a_i, b_i, t_i\}$ for all $i \in [n]$. Then, define the edge set $E' \coloneqq B \cup D_1 \cup D_2 \cup E_1 \cup E_2$ comprising

$$\begin{aligned} B &\coloneqq \{\{a_i, b_i\} \mid i \in [n]\}, \\ D_1 &\coloneqq \{\{s_i, a_i\} \mid i \in [n]\}, \\ D_2 &\coloneqq \{\{b_i, t_i\} \mid i \in [n]\}, \\ E_1 &\coloneqq \{\{s_i, a_j\} \mid \{i, j\} \in E\}, \text{ and} \\ E_2 &\coloneqq \{\{b_i, t_j\} \mid \{i, j\} \in E\}. \end{aligned}$$

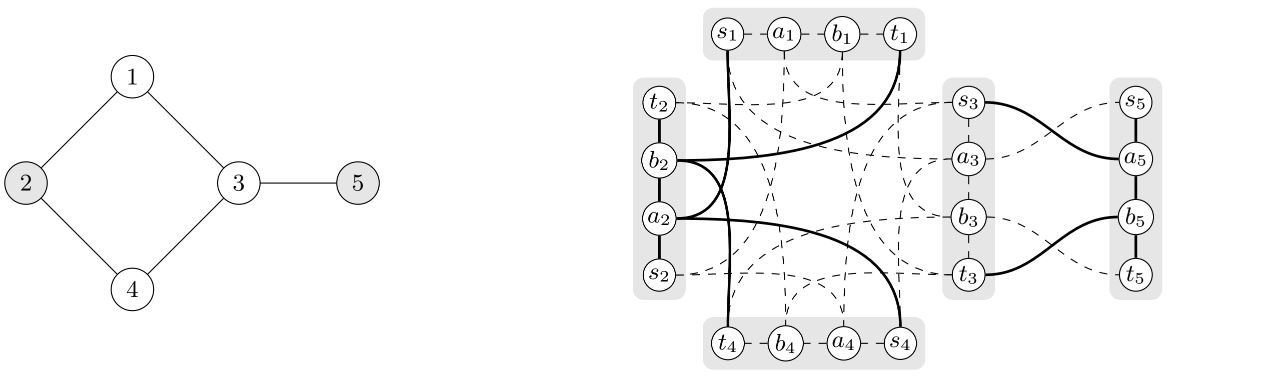

(a) A positive instance of IDS with $|V| = 5$ and $k = 2$; a solution $U = \{2, 5\}$ is drawn in gray.

(b) An instance of SRN; the establishing paths of a solution π starting with $\{s_2, t_2\}$ and $\{s_5, t_5\}$ are drawn as solid lines. Edge costs not shown.

Fig. S1. Polynomial time reduction from IDS to SRN. For $\{i, j\} \in E$, the path (s_i, a_j, b_j, t_i) is cheaper than (s_i, a_i, b_i, t_i) if and only if (s_j, a_j, b_j, t_j) was used before, encoding the selection of j into an independent dominating set.

We refer to edges in B as *bridges* (since any s_i-t_i-path needs to contain such an edge) and we can think of the sets D_1 and D_2 as *direct* edges (remaining within V_i for some $i \in [n]$) and of the sets E_1 and E_2 as *external* edges. Next, let $\beta \in \mathbb{Z}$ be an arbitrary integer strictly larger than $\frac{2}{1-\alpha}$, so that $3\beta - 2 > (\alpha + 2)\beta$, and use it to define the edge costs $c(e) := \beta$ for all $e \in B \cup E_1 \cup E_2$ and $c(e) := \beta - 1$ for all $e \in D_1 \cup D_2$ (direct edges are initially cheaper than external ones). Lastly, define the set of connections $K := \{\{s_i, t_i\} \mid i \in [n]\}$ and set the target social cost to

$$\gamma := k\,(3\beta - 2) + (n - k)(\alpha + 2)\beta.$$

Intuitively, we are looking to establish a one-to-one correspondence between $\{a_i, b_i\}$-edges appearing in π-establishing paths and the corresponding vertex i being selected into the independent dominating set $U \subseteq V$. It is obvious that the reduction can be computed in polynomial time. Further, all assigned costs are positive integers as required.

Whenever we consider a connection order $\pi \in S(K)$ in the following, we assume without loss of generality that the vertices in V are labeled according to this order, that is $\pi = (\{s_i, t_i\})_{i=1}^{n}$. This allows us to identify a time $i \in [n]$ with a vertex $i \in V$ whose corresponding connection $\{s_i, t_i\} \in K$ is established at that time. For brevity, we further write $c_i(e)$ for the reduced cost of an edge e at time i, formally $c_i(e) := r(c(e), \tau_e(i))$ where $\tau_e(i)$ is the tier of e at time i.

Denote by $B_i = \{j \in [n] \mid \{a_j, b_j\}$ is i-cheap$\}$ the indices of bridges lying on some establishing path prior to time i. Before we prove decision equivalence, we show by induction that at every time $i \in [n]$, the path establishing π_i is of the form (s_i, a_j, b_j, t_i) for some $j \in N(i) \cap B_i$, if this intersection is nonempty, and $j = i$, otherwise. At time $i = 1$, we have $N(1) \cap B_1 = \emptyset$ as all edges are tier zero and (s_i, a_i, b_i, t_i) is the unique shortest s_i-t_i-path by construction.

At a time $i \in [n]$ with $i > 1$, first consider the case of $N(i) \cap B_i \neq \emptyset$ and let $j \in N(i) \cap B_i$. By induction assumption, the edges $\{s_i, a_j\}$ and $\{b_j, t_i\}$ are i-costly, so the path (s_i, a_j, b_j, t_i) has cost $\beta + \alpha\beta + \beta = (\alpha + 2)\beta$. On the other hand, this value is a lower bound for the cost of any path P not remaining in V_i, as leaving from V_i and returning to V_i both incur a cost of at least β, and the cost for crossing a bridge is at least $\alpha\beta$, hence $c_i(P) \geq (\alpha + 2)\beta$. The only path remaining in V_i is (s_i, a_i, b_i, t_i) with cost $(\beta - 1) + \beta + (\beta - 1) = 3\beta - 2 > (\alpha + 2)\beta$, showing that (s_i, a_j, b_j, t_i) is a least-cost s_i-t_i-path at time i.

Consider next the case $N(i) \cap B_i = \emptyset$, that is, for all $j \in N(i)$ the bridge $\{a_j, b_j\}$ is i-costly. Let P be an arbitrary path *not using* the bridge $\{a_i, b_i\}$. If P uses a bridge $\{a_j, b_j\}$ for some $j \in N(i)$, then $c_i(P) \geq 3\beta > 3\beta - 2$. Otherwise, $P = (e_1, e_2, \ldots, e_{|P|})$ contains at least 5 distinct edges. Since the first bridge of P is not indexed in $N(i) \cup \{i\}$, the first two edges e_1 and e_2 of P are both in $D_1 \cup E_1$, and at most one of them is in D_1. Moreover, by induction assumption, these two edges must be i-costly, hence $c_i(e_1) + c_i(e_2) \geq \beta + (\beta - 1) = 2\beta - 1$. A symmetric reasoning over the last two edges of P shows $c_i(e_{|P|-1}) + c_i(e_{|P|}) \geq 2\beta - 1$ and thus $c_i(P) \geq 4\beta - 2 > 3\beta - 2$. This shows that the path (s_i, a_i, b_i, t_i) is the unique least-cost s_i-t_i-path, which concludes the induction.

Now that we have circumscribed the possible π-establishing paths for any $\pi \in S(K)$, we will establish their reduced costs. Let $\pi \in S(K)$ be fixed and consider the path establishing π_i for some $i \in [n]$. If the path is (s_i, a_i, b_i, t_i), then all its edges are i-costly and it has a reduced cost of

$$r(c(\{s_i, a_i\}), 0) + r(c(\{a_i, b_i\}), 0) + r(c(\{b_i, t_i\}), 0) = 3\beta - 2.$$

We call such a path an *independent path.* Otherwise, π_i is established by a path (s_i, a_j, b_j, t_i), and $\{a_j, b_j\}$ is i-cheap. Thus, (s_i, a_j, b_j, t_i) has a reduced cost of

$$r(c(\{s_i, a_j\}), 0) + r(c(\{a_j, b_j\}), \tau) + r(c(\{b_j, t_i\}), 0) = (\alpha + 2)\beta$$

where $\tau \in \mathbb{Z}_{\geq 1}$ is the tier of $\{a_j, b_j\}$ at time i. We call such a path a *dominated path.* In total, the social cost of π is

$$k'\,(3\beta - 2) + (n - k')(\alpha + 2)\beta$$

where $k' \in [n]$ is the number of independent paths establishing π.

We are now prepared to show decision equivalence. For a first direction, let $U \subseteq V$ with $|U| \leq k$ be an independent dominating set. We construct a connection order π starting from an empty sequence as follows: First, for every $i \in U$ in any order, append $\{s_i, t_i\} \in K$ to π. Then, for every $j \in V \setminus U$ in any order, append $\{s_j, t_j\} \in K$ to π. We show that $C(\pi) \leq \gamma$. Recall that we assume that π matches the vertex labels such that $U = [k]$ and $V \setminus U = [n] \setminus [k]$. Since U is an independent set, we have for every $i \in [k]$ and every $h \in [i-1] \subseteq U$ that there is no edge $e \in E'$ connecting a vertex in V_h with one in V_i. Hence, π_i is established by an independent path for every $i \in [k]$. Since U is also a dominating set, we have for every $j \in [n]$ with $j > k$ that there is some $i \in [k]$ such that $\{i, j\} \in E$. Thus, π_j is established by a dominated path and we have

$$C(\pi) = k\,(3\beta - 2) + (n-k)(\alpha+2)\beta = \gamma.$$

For the reverse direction, let $\pi \in S(K)$ such that $C(\pi) \leq \gamma$. We construct an independent dominating set $U \subseteq V$ as follows: For every $i \in [n]$, add i to U if and only if π_i is established by an independent path. We first show that $|U| \leq k$. Assume towards a contradiction that $|U| > k$. Then, at least $k+1$ π-establishing paths are independent, and thereby at most $n-k-1$ π-establishing paths are dominated. As independent paths have a larger reduced cost than dominated paths, this gives

$$C(\pi) \geq (k+1)\,(3\beta - 2) + (n-k-1)(\alpha+2)\beta = \gamma + (3\beta - 2) - (\alpha+2)\beta > \gamma,$$

where the last inequality follows from our definition of β, contradicting $C(\pi) \leq \gamma$. We next show that U is an independent set. Assume towards a contradiction that there are $i, j \in U$ with $\{i, j\} \in E$. Without loss of generality let $i < j$; the case of $j < i$ is analogous with i and j swapped. Then, π_j was established by a dominated path, contradicting $j \in U$. Last we show that U is a also a dominating set. Assume towards a contradiction that there is some $i \in V \setminus U$ such that there is no $j \in U$ with $\{i, j\} \in E$. Since $i \notin U$, π_i must be established by a dominated path. Hence, there must be some $h \in [i]$ such that π_h was established by an independent path and $\{h, i\} \in E$. The case of $h \in V \setminus U$ contradicts the definition of U while the case of $h \in U$ contradicts the assumption that there is no $j \in U$ with $\{i, j\} \in E$. In summary, we have that U is an independent dominating set with $|U| \leq k$. ☐

Corollary 1. *`SRN` with $\alpha < 1$ remains NP-complete on networks with unit edge costs and vertex degree at most five.*

Proof. For the bound on the degree, note that `IDS` is already hard on cubic graphs which are reduced to an instance of `SRN` where vertices have a degree of either four or five by construction. For unit edge costs, replace each edge in the resulting network with a path whose topological length is the integral cost of the edge being replaced. ☐

Corollary 2. *There exists no polynomial-time $1 + (1-\epsilon)\frac{(1-\alpha)}{8160}$-approximation algorithm for `MIN-SRN` with $\alpha < 1$ for any $\epsilon > 0$, unless $P = NP$.*

Proof. Let $\alpha \in [0, 1)$ be fixed, define $\omega := 1 - \alpha > 0$ for brevity, and adapt the reduction in the proof of Theorem 1 as follows. From the given instance of `IDS`, strip the integer k and require the graph $G = (V, E)$ to have maximum degree $\Delta = 3$. Consider then $I := (G)$ an instance of `MIN-IDS`. From the reduction procedure, strike the definition of the target cost γ and denote the remainder formally by a function f that maps an instance of `MIN-IDS` to an instance (N, K, α) of `MIN-SRN` where N is a network and K a set of connections. Recall that the edge costs of N depend on a constant β and set it equal to $\lceil \frac{2}{\omega} \rceil + \kappa$ for some integer $\kappa > (\frac{1}{\epsilon} - 1)(\frac{2}{\omega} + 1)$, so that

$$(3\beta - 2) - (\alpha+2)\beta = \omega\beta - 2 \in [\omega\kappa,\ \omega(\kappa+1)).$$

For a given `MIN-IDS` instance $\bar{I} = (\bar{G})$ with $\bar{G} = (\bar{V}, \bar{E})$, let further g be a function that maps a feasible solution π of $f(\bar{I}) = (\bar{N}, \bar{K}, \alpha)$ to a solution U of $\bar{I}$, as follows: $U := g(\pi)$ is the set of vertices $i \in \bar{V}$ where the path establishing the connection $\{s_i, t_i\} \in \bar{K}$ in the formation of $R_{\bar{N}, r_\alpha}(\pi)$ is the independent path (s_i, a_i, b_i, t_i). It is clear that f and g can be computed in polynomial time.

We show that (f, g) is an L-reduction from `MIN-IDS` to `MIN-SRN`. To this end let k^* denote the independent domination number of G and let π^* be an optimum solution to $f(I)$. From the proof of Theorem 1 it can be seen that a feasible solution of size k to I implies a feasible solution of social cost $\Gamma(k) := k\,(3\beta - 2) + (n-k)(\alpha+2)\beta$ to $f(I)$ and, vice versa, a feasible solution π to $f(I)$ has a social cost of $\Gamma(k)$ for some k and $g(\pi)$ is a feasible solution to I of size k. Thus, we have for the optimum values k^* of I and $C(\pi^*)$ of $f(I)$ that

$$\begin{aligned} C(\pi^*) &= k^*\,(3\beta - 2) + (n-k^*)(\alpha+2)\beta \\ &= n(\alpha+2)\beta + k^*(\omega\beta - 2) \\ &\leq (1+\Delta)k^*(\alpha+2)\beta + k^*(\omega\beta - 2) \\ &= (4(\alpha+2)\beta + \omega\beta - 2)k^* \\ &= (12\beta - 3\omega\beta - 2)\,k^* \\ &< \left(\frac{24}{\omega} + 12(\kappa+1) - 3\omega\beta - 2\right)k^* \\ &< 12\left(\frac{2}{\omega} + \kappa + 1\right)k^* \end{aligned}$$

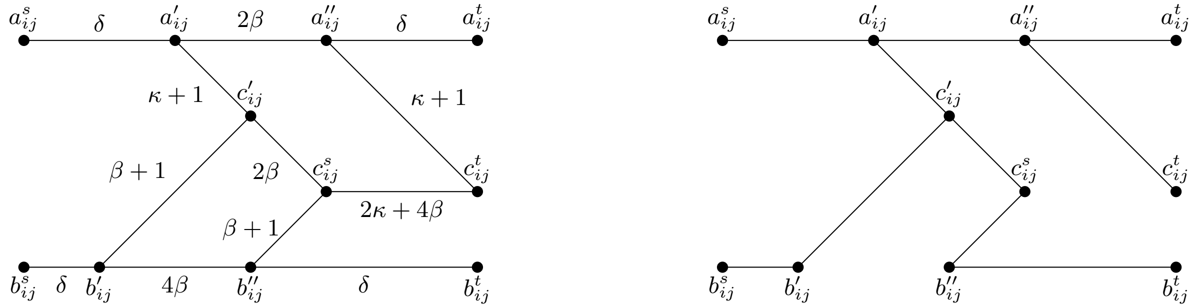

(a) The gadget G_{ij} with initial edge costs. For all $x \in \{a, b, c\}$, some establishing path contains an x_{ij}^s-x_{ij}^t-path as a subpath.

(b) The target gadget $G_{ij}[T]$. It is induced, $G_{ij}[F] = G_{ij}[T]$, iff the connection order obeys $\{a_{ij}^s, a_{ij}^t\} \prec \{c_{ij}^s, c_{ij}^t\} \prec \{b_{ij}^s, b_{ij}^t\}$.

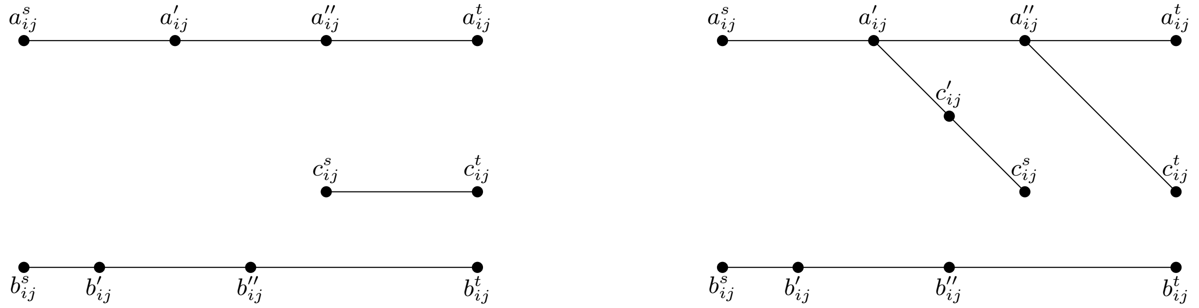

(c) $G_{ij}[F]$ induced by all connection orders with $\{c_{ij}^s, c_{ij}^t\} \prec \{a_{ij}^s, a_{ij}^t\}$.

(d) $G_{ij}[F]$ induced by all connection orders with $\{a_{ij}^s, a_{ij}^t\}, \{b_{ij}^s, b_{ij}^t\} \prec \{c_{ij}^s, c_{ij}^t\}$.

Fig. S2. A network gadget representing a constraint of the Arity 2 Permutation CSP problem. Note that we simplify notation by identifying $\{x_{ij}^s, x_{ij}^t\}$ for $x \in \{a, b, c\}$ with a connection whose establishing path contains both vertices.

where the first inequality follows from $\left\lceil \frac{n}{1+\Delta} \right\rceil \leq k^*$ (4) while the second inequality holds due to $\omega\beta - 2 < \omega(\kappa + 1)$ and the last one simply follows from $3\omega\beta + 2 > 0$. Furthermore, we have for a feasible solution π to $f(I)$ with $k := |g(\pi)|$ that

$$|C(\pi^*) - C(\pi)| = |n(\alpha+2)\beta + k^*(\omega\beta - 2) - (n(\alpha+2)\beta + k(\omega\beta - 2))| = (\omega\beta - 2)\,|k^* - k|\,.$$

Since $\omega\beta - 2 \geq \omega\kappa$, we can relate the suboptimalities of π and $g(\pi)$ by

$$|k^* - k| \leq \frac{1}{\omega\kappa}|C(\pi^*) - C(\pi)|.$$

Therefore, (f, g) is an L-reduction with parameters $12\left(\frac{2}{\omega} + \kappa + 1\right)$ and $\frac{1}{\omega\kappa}$. This means that the existence of a $(1+\rho)$-approximation algorithm for `MIN-SRN` implies that there exists a $1 + \frac{12}{\omega\kappa}(\frac{2}{\omega} + \kappa + 1)(\rho - 1)$-approximation algorithm for `MIN-IDS`; this was shown for instance in (5).

Now, assume that there exists a $1 + \frac{(1-\epsilon)\omega}{8160}$-approximation algorithm for `MIN-SRN`. The L-reduction implies the existence of an approximation algorithm for `MIN-IDS` with performance guarantee

$$\begin{aligned} 1 + \frac{12}{\omega\kappa}\left(\frac{2}{\omega} + \kappa + 1\right)\frac{(1-\epsilon)\omega}{8160} &= 1 + \frac{1-\epsilon}{680}\left(1 + \frac{1}{\kappa}(\frac{2}{\omega} + 1)\right) \\ &< 1 + \frac{1-\epsilon}{680}\left(1 + \frac{\epsilon}{1-\epsilon}\right) \\ &= 1 + \frac{1}{680}, \end{aligned}$$

where the inequality follows from our choice of κ. By Fact 2, this implies P = NP. ☐

Theorem 2. *SRN-FIT with $\alpha \in \left[\frac{1}{2}, 1\right)$ and $d := d_w$ is NP-complete even for integral base costs $c(E) \subseteq \mathbb{Z}_{\geq 1}$ and uniform weights $w(E) = \{1\}$.*

Proof. Containment in NP follows from the fact that the procedure in Definition 1 (producing the edge set F) and the resulting difference $d_w(G[F], G[T]) = |(T \setminus F) \cup (F \setminus T)|$ can be computed in polynomial time.

To show NP-hardness, we present a polynomial-time reduction from Arity 2 Permutation CSP. Let thus a ground set U, a set of constraints $C \subseteq U^2$, and an integer $k \in \mathbb{Z}_{\geq 0}$ compose an instance of Arity 2 Permutation CSP. We assume without loss of generality that the ground set contains no unconstrained elements, formally $\forall u \in U \colon \exists v \in U \colon (u, v) \in C \vee (v, u) \in C$,

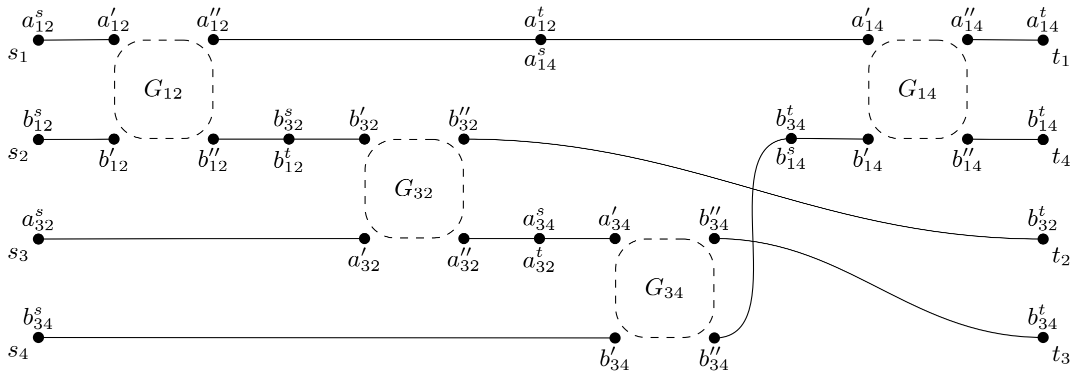

Fig. S3. A network representing an instance of ARITY 2 PERMUTATION CSP with $U = [4]$ and $C = \{(1,2),(3,2),(3,4),(1,4)\}$. Gadgets G_{ij} with $i > j$ are flipped vertically compared to Figure S2. Doubly labeled vertices are identified.

and further that no pair of conflicting constraints is present, that is $\forall(u,v) \in C\colon (v,u) \notin C$. In the former case, unconstrained elements can be ordered arbitrarily without affecting the number of fulfilled constraints while in the latter case one can remove both constraints and decrement k. We construct a decision-equivalent instance of `SRN-FIT` from an empty network $N = (V, E, c)$ as follows: First, we define the integer costs $\beta := \lceil \frac{1}{1-\alpha} \rceil + 1$, $\delta := 2\beta + 12$, and $\kappa := |U|\alpha^{-1}(2\delta + 4\beta)$. This choice gives $\alpha\beta \in (\beta - 3, \beta - 1)$ as

$$\alpha\beta \geq \alpha\left(\frac{1}{1-\alpha} + 1\right) > \frac{\alpha}{1-\alpha} = \frac{1}{1-\alpha} - 1 > \left\lceil \frac{1}{1-\alpha} \right\rceil - 2 = \beta - 3,$$
$$\beta > \frac{1}{1-\alpha} \implies (1-\alpha)\beta > 1 \implies \beta - \alpha\beta > 1 \implies \alpha\beta < \beta - 1$$

and further $\alpha\delta > 2\beta$ due to the above and $\alpha \geq \frac{1}{2}$. Then, for every constraint $(i,j) \in C$, we add a gadget $G_{ij} = (V_{ij}, E_{ij})$ as shown in Figure S2a to N, formally

$$V_{ij} := \{a^s_{ij}, a'_{ij}, a''_{ij}, a^t_{ij}\} \cup \{b^s_{ij}, b'_{ij}, b''_{ij}, b^t_{ij}\} \cup \{c^s_{ij}, c'_{ij}, c^t_{ij}\} \text{ and}$$
$$E_{ij} := A_{ij} \cup B_{ij} \cup K_{ij}$$

where

$$A_{ij} := \left\{\{a^s_{ij}, a'_{ij}\}, \{a'_{ij}, a''_{ij}\}, \{a''_{ij}, a^t_{ij}\}\right\},$$
$$B_{ij} := \left\{\{b^s_{ij}, b'_{ij}\}, \{b'_{ij}, b''_{ij}\}, \{b''_{ij}, b^t_{ij}\}\right\} \cup \left\{\{b'_{ij}, c'_{ij}\}, \{c'_{ij}, c^s_{ij}\}, \{c^s_{ij}, b''_{ij}\}\right\}, \text{ and}$$
$$K_{ij} := \left\{\{c^s_{ij}, c^t_{ij}\}, \{a'_{ij}, c'_{ij}\}, \{a''_{ij}, c^t_{ij}\}\right\}$$

and with edge base costs given by

$$c\left(\left\{\{a^s_{ij}, a'_{ij}\}, \{a''_{ij}, a^t_{ij}\}, \{b^s_{ij}, b'_{ij}\}, \{b''_{ij}, b^t_{ij}\}\right\}\right) := \{\delta\},$$
$$c\left(\left\{\{a'_{ij}, c'_{ij}\}, \{a''_{ij}, c^t_{ij}\}\right\}\right) := \{\kappa + 1\},$$
$$c\left(\left\{\{a'_{ij}, a''_{ij}\}, \{c^s_{ij}, c'_{ij}\}\right\}\right) := \{2\beta\},$$
$$c\left(\{c^s_{ij}, c^t_{ij}\}\right) := 2\kappa + 4\beta,$$
$$c\left(\{b'_{ij}, b''_{ij}\}\right) := 4\beta.$$

Additionally, we add isolated vertices s_i and t_i for every $i \in U$. It may be helpful to note that K_{ij} contains precisely the edges with a cost of at least κ and that $G_{ij}[E_{ij} \setminus K_{ij}]$ has connected components $G_{ij}[A_{ij}]$ and $G_{ij}[B_{ij}]$.

We wire the gadgets as follows (see Figure S3 for an example): First, fix an arbitrary order on the constraints C. Then, for every $i \in U$, let $C_{i\bullet} := \{(i,j) \in C \mid j \in U\}$, $C_{\bullet i} := \{(j,i) \in C \mid j \in U\}$, and $C_i := C_{i\bullet} \cup C_{\bullet i}$ be sets of constraints involving i. For every $\ell \in [\,|C_i|\,]$ in order, we consider the ℓ-th constraint x^ℓ from $C_i \subseteq C$ according to our ordering of C and we let both $s^\ell := a^s_{ij}$ and $t^\ell := a^t_{ij}$, if $x^\ell = (i,j) \in C_{i\bullet}$, or both $s^\ell := b^s_{ji}$ and $t^\ell := b^t_{ji}$, if $x^\ell = (j,i) \in C_{\bullet i}$. Further, we identify s^ℓ with s_i, if $\ell = 1$, s^ℓ with $t^{\ell-1}$, whenever $\ell > 1$, and t^ℓ with t_i, if $\ell = |C_i|$. Lastly, we define the set of connections

$$K := \{\{s_i, t_i\} \mid i \in U\} \cup \left\{\{c^s_{ij}, c^t_{ij}\} \mid (i,j) \in C\right\}$$

and set the target distance to $\gamma := 3\,(|C| - k)$ and the target edge set (Figure S2b) to

$$T := \bigcup_{(i,j)\in C} \left(E_{ij} \setminus \left\{\{b'_{ij}, b''_{ij}\}, \{c^s_{ij}, c^t_{ij}\}\right\}\right).$$

It is obvious that the reduction can be computed in polynomial time. Further, all assigned costs are positive integers as required.

Informally, every element $i \in U$ is now associated with a connection $\{s_i, t_i\} \in K$ and by extension with a shortest (with respect to base costs) s_i-t_i-path that traverses precisely the gadgets that correspond to constraints involving i. Indeed, we will later show that the establishing path for such a connection will not be too different from this initial shortest path. The remaining connections, which are $\{c_{ij}^s, c_{ij}^s\}$ for all $(i,j) \in C$, allow the gadget G_{ij} to detect the relative order of $\{s_i, t_i\}$ and $\{s_j, t_j\}$. More precisely, fulfilling (i,j) corresponds to ordering the associated connections as $\{s_i, t_i\} \prec \{c_{ij}^s, c_{ij}^t\} \prec \{s_j, t_j\}$.

Let us now analyze the instance of SRN-FIT constructed above. First, we investigate what establishing paths can occur in principle. To this end we define the sets

$$E_i := \left(\bigcup_{(i,j) \in C_{i\bullet}} A_{ij} \right) \cup \left(\bigcup_{(j,i) \in C_{\bullet i}} B_{ji} \right) \qquad \text{and} \qquad V_i := \bigcup_{e \in E_i} e$$

for every $i \in U$ and show two related claims in the following.

Claim 1. A path P_i establishing the connection $\{s_i, t_i\}$ is contained in $G[E_i]$.

Proof of Claim 1. By construction, any other s_i-t_i-path must use an edge e with a base cost of $c(e) = \kappa + 1$. However, it is $\alpha(\kappa + 1) > \alpha\kappa = |U|(2\delta + 4\beta)$ with the right hand side posing an upper bound on the least cost of an s_i-t_i-path at any time.

Claim 2. A path P_{ij} establishing the connection $\{c_{ij}^s, c_{ij}^t\}$ for some $(i,j) \in C$ is contained within G_{ij}.

Proof of Claim 2. By induction on the time t. Let t_0 be the first time where a connection $\pi_{t_0} = \{c_{ij}^s, c_{ij}^t\}$ with $(i,j) \in C$ is established by a path P_{ij}. Then, any connection established at a time $t < t_0$ has the form $\{s_\ell, t_\ell\}$ with $\ell \in U$. By Claim 1, none of these earlier connections is established by a path using an edge with base cost at least κ, so any such edge has also a reduced cost of at least κ at time t_0. Assume now towards a contradiction that P_{ij} leaves the gadget G_{ij}. By construction, P_{ij} must then contain at least one of the two edges incident to c_{ij}^t, both of which have a reduced cost of at least κ at time t_0, and P_{ij} must further use at least two distinct vertices $u, v \in \{a_{ij}^s, a_{ij}^t, b_{ij}^s, b_{ij}^t\}$ on the "boundary" of G_{ij}. We distinguish two cases. If $\{u, v\} = \{a_{ij}^s, a_{ij}^t\}$ (respectively $\{u, v\} = \{b_{ij}^s, b_{ij}^t\}$), then P_{ij} contains an a_{ij}^s-a_{ij}^t-subpath (a b_{ij}^s-b_{ij}^t-subpath) P'_{ij} containing at least two edges from $E \setminus E_{ij}$. Note that P'_{ij} cannot be completely contained within the set E_i (respectively E_j) as its endpoints form a cut set that partitions $G[E_i]$ ($G[E_j]$) into three components, two distinct of which contain, respectively, the second and penultimate vertex of P'_{ij}. Thus, P'_{ij} must contain at least two distinct edges with precisely one endpoint in V_i and in $V \setminus V_i$ each, none of which can be incident to c_{ij}^t and both of which have a reduced cost of at least κ at time t_0 by construction. The path P_{ij} has thereby a reduced cost of at least $3\kappa > 2\kappa + 4\beta = r(\{c_{ij}^s, c_{ij}^t\}, 0)$ at time t_0, contradicting its minimality. In the remaining case of $\{u, v\} \in \{\{a_{ij}^s, b_{ij}^s\}, \{a_{ij}^t, b_{ij}^t\}, \{a_{ij}^s, b_{ij}^t\}, \{a_{ij}^t, b_{ij}^s\}\}$, P_{ij} contains a vertex in V_i and a vertex in V_j. It must thus contain a subpath P'_{ij} with edges disjoint from E_{ij} that contains precisely one vertex in V_i and in V_j each. By construction, no edge outside of E_{ij} has one endpoint in each of V_i and in V_j (otherwise it must be the case that both $(i,j) \in C$ and $(j,i) \in C$). It follows that P'_{ij} must contain another subpath P''_{ij} contained in V_ℓ for some $\ell \in U \setminus \{i, j\}$. As in the first case, two edges with a reduced cost of at least κ are necessary to enter and leave V_ℓ, again contradicting the minimality of P_{ij}. This concludes the induction basis. The argument for the induction step is analogous with two modifications: When a connection $\pi_t = \{c_{ij}^s, c_{ij}^t\}$ with $(i,j) \in C$ is established at some time $t > t_0$, then the induction assumption in combination with Claim 1 again ensures that all edges in E_{ij} with base cost at least κ also have a reduced cost of at least κ at time t. However, any edge $e \in E \setminus E_{ij}$ with base cost $c(e) \geq \kappa$ can, in principal, have a reduced cost of only $\alpha c(e)$ at time t. Thus, a path P_{ij} that establishes π_t and leaves G_{ij} has a reduced cost of at least $(\kappa + 1) + 2\alpha\kappa > 2\kappa$. Adding to this a lower bound for the reduced costs of cheaper edges not counted so far, namely $2\alpha\delta > 4\beta$ for two edges incident to distinct vertices in $\{a_{ij}^s, a_{ij}^t, b_{ij}^s, b_{ij}^t\}$, we arrive at a reduced cost larger than $2\kappa + 4\beta = r(\{c_{ij}^s, c_{ij}^t\}, 0)$, contradicting again the minimality of P_{ij}. This proves the second claim.

As all edges of G are contained in gadgets, we may write the total disutility for the constructed instance as the sum

$$d_w(G[F], G[T]) = |(T \setminus F) \cup (F \setminus T)| = \sum_{(i,j) \in C} |(T_{ij} \setminus F_{ij}) \cup (F_{ij} \setminus T_{ij})|$$

where $T_{ij} := T \cap E_{ij}$ are the target edges of G_{ij} and where F_{ij} are the edges of π-establishing paths within G_{ij} for a solution candidate $\pi \in S(K)$. Let us thus fix a constraint $(i,j) \in C$ and determine F_{ij}. From Claims 1 and 2 and from the definition of the sets E_i and E_j, it follows that precisely three connections have π-establishing paths that traverse G_{ij}, being $A := \{s_i, t_i\}$, $B := \{s_j, t_j\}$ and $C := \{c_{ij}^s, c_{ij}^t\}$. Further, the path establishing A induces an a_{ij}^s-a_{ij}^t-subpath in G_{ij} and the path establishing B induces an b_{ij}^s-b_{ij}^t-subpath in G_{ij}. We distinguish three cases with respect to the relative order of $A, B, C \in K$ within π. It might be helpful to recall that we chose edge costs such that $\beta - 3 < \alpha\beta < \beta - 1$ and $\alpha\kappa = |U|(2\delta + 4\beta)$.

Case 1: $A \prec C \prec B$**.** The path establishing A at some time t_0 is the first path to traverse G_{ij} and does so via a subpath $(a_{ij}^s, a'_{ij}, a''_{ij}, a_{ij}^t)$ with reduced cost $2\delta + 2\beta < \kappa + 1$. When C is established at a time $t_1 > t_0$, then the path $:= (c_{ij}^s, c'_{ij}, a'_{ij}, a''_{ij}, c_{ij}^t)$ has a reduced cost of $2\beta + 2(\kappa + 1) + \alpha(2\beta) < 2\kappa + 4\beta$. By Claim 2 and the construction of G_{ij}, this path thus establishes C. Finally, when B is established at a time $t_2 > t_1$, then due to $2(\beta + 1) + \alpha(2\beta) < 2(\beta + 1) + 2(\beta - 1) = 4\beta$ (and $\alpha(\kappa + 1) > \alpha(2\beta)$), the establishing path induces the subpath $(b_{ij}^s, b'_{ij}, c'_{ij}, c_{ij}^s, b''_{ij}, b_{ij}^t)$ in G_{ij}. It follows that $F_{ij} = T_{ij}$, so in this case the disutility contributed by G_{ij} is 0.

Case 2: $C \prec A$**.** This includes three subcases: $B \prec C \prec A$ and $C \prec B \prec A$ and $C \prec A \prec B$. First note that the path establishing A unconditionally induces the subpath $P_A := (a_{ij}^s, a'_{ij}, a''_{ij}, a_{ij}^t)$ in G_{ij} as $2\delta + 2\beta < \alpha(\kappa + 1)$. For the

path establishing B, consider the critical edge set $X \coloneqq \{\{b'_{ij}, c'_{ij}\}, \{c'_{ij}, c^s_{ij}\}, \{c^s_{ij}, b''_{ij}\}\}$: If all edges in X have a reduced cost equal to their initial cost at the time when B is established, then clearly the path establishing B induces the subpath $(b^s_{ij}, b'_{ij}, c'_{ij}, b^t_{ij})$ in G_{ij}. If $B \prec C \prec A$, then this condition is trivially satisfied and further C is established by the path (c^s_{ij}, c^t_{ij}) as $2\kappa + 4\beta < 2(\kappa+1) + 2(2\beta)$ and $2\beta < 2(\beta+1)$, ruling out both alternative paths that do not leave G_{ij}. If $C \prec A, B$, then C is again established by the path (c^s_{ij}, c^t_{ij}) disjoint from X. Further, the unconditional path P_A is disjoint from X. So in all subcases the intersection of π-establishing paths with E_{ij} is equivalent and includes the non-target edges $\{b'_{ij}, b''_{ij}\}$ and $\{c^s_{ij}, c^t_{ij}\}$ while excluding the target edges $\{a'_{ij}, c'_{ij}\}$, $\{c^t_{ij}, a''_{ij}\}$, $\{b'_{ij}, c'_{ij}\}$, and $\{c^s_{ij}, b''_{ij}\}$. Thus, G_{ij} contributes a disutility of 6 in this case.

Case 3: $A, B \prec C$**.** As for Case 2, A is unconditionally established by a path inducing in G_{ij} the subpath P_A disjoint from X (both as defined there) and therefore B is established with the same subpath as in Case 2. Further C is established as in Case 1. The intersection of π-establishing paths with E_{ij} thus includes the non-target edge $\{b'_{ij}, b''_{ij}\}$ and excludes the targe edges $\{b'_{ij}, c'_{ij}\}$ and $\{c^s_{ij}, b''_{ij}\}$. Thus, G_{ij} contributes a disutility of 3 in this case.

We can now prove decision equivalence. Let first the constructed instance of `SRN-FIT` be a *yes*-instance with solution $\pi \in S(K)$. We construct a solution π' to the associated instance of Arity 2 Permutation CSP as follows. Without loss of generality we may assume that Case 2 does not occur for any gadget: Otherwise, for a constraint $(i,j) \in C$ and associated connections $A \coloneqq \{s_i, t_i\}$, $B \coloneqq \{s_j, t_j\}$ and $C \coloneqq \{c^s_{ij}, c^t_{ij}\}$ for which Case 2 occurs, we can make G_{ij} contribute less disutility (either 3 or 0 instead of 6) by moving C such that $A \prec C$. Clearly this does not affect the subpaths induced by π-establishing paths within any other gadget so that the total disutility decreases by this change. Thus, for every $(i,j) \in C$, the gadget G_{ij} contributes either no disutility, if $\{s_i, t_i\} \prec_\pi \{s_j, t_j\}$, or a disutility of 3, otherwise. It follows immediately that the solution $\pi' \in S(U)$ corresponding to the total order $\big\{(i,j) \in U^2 \mid \{s_i, t_i\} \prec_\pi \{s_j, t_j\}\big\}$ violates at most $\frac{\gamma}{3}$ constraints, thereby fulfilling at least $|C| - \frac{\gamma}{3} = k$ constraints.

Let last the instance of Arity 2 Permutation CSP be a *yes*-instance with solution $\pi' \in S(U)$ and define the relation

$$R \coloneqq \Big\{(\{s_i, t_i\}, \{c^s_{ij}, c^t_{ij}\}) \mid (i,j) \in C\Big\} \cup \Big\{(\{c^s_{ij}, c^t_{ij}\}, \{s_j, t_j\}) \mid (i,j) \in C \wedge i \prec_{\pi'} j\Big\} \subseteq K^2.$$

As we assume that $\forall (i,j) \in C\colon (j,i) \notin C$ and since $\prec_{\pi'}$ is an order, we have that R is antisymmetric and hence can be extended to a total order on K. Let thus $\pi \in S(K)$ such that $\prec_\pi \supseteq R$. To show that π is a solution to the constructed instance of `SRN-FIT`, let $(i,j) \in C$ and consider first the case where $i \prec_{\pi'} j$ (C is fulfilled). Then, we have $\{s_i, t_i\} \prec_\pi \{c^s_{ij}, c^t_{ij}\} \prec_\pi \{s_j, t_j\}$ and so the gadget G_{ij} associated with (i,j) contributes no disutility (Case 1). Consider next the case where $j \prec_{\pi'} i$ (C is violated). Then, it is $\{s_i, t_i\} \prec_\pi \{c^s_{ij}, c^t_{ij}\}$ and so G_{ij} contributes a disutility of at most 3 (Case 1 or 3). As π' violates at most $|C| - k$ constraints, the disutility of π is at most $3(|C| - k) = \gamma$. □

Procedures.

Detailed version of the construction of the Euclidean embedding. Consider the procedure in Definition 1 with r, N, and K with $k = |K|$ all fixed. Let D be a symmetric, nonnegative, zero-trace $k \times k$ matrix such that D_{ij} denotes the expected dissimilarity between $R(\pi)$ and $R(\pi^{ij})$ where π^{ij} is equal to π with the i-th and j-th positions exchanged and where π is drawn uniformly from $S(K)$. Let d be the superdiagonal of D and assume that d is strictly monotonic. (In practice, fit a strictly monotonic function f to d.) Assume further that $D_{ij} = D_{i\ell} + D_{\ell j}$ for all $i < \ell < j$, as was empirically observed for $r = r_\alpha$. Then, a transformation $\Phi\colon S(K) \to \mathbb{R}^{k-1}$ with the property that $\|\Phi(\pi) - \Phi(\pi^{ij})\| = D_{ij}$ holds for all $\pi \in S(K)$ and $i, j \in [k]$ is obtained from d as follows.

First, define $w = \|w'\|_1^{-1} w' \in \mathbb{R}^k_{>0}$ recursively via

$$w'_i = \begin{cases} 1, & \text{if } i = 1, \\ w'_{i-1} e^{\frac{1}{\sqrt{2}} d_{i-1}}, & \text{if } 1 < i \le k, \end{cases}$$

and let w^{ij} denote w with the i-th and j-th entry exchanged. Then, it is $\log \frac{w_{i+1}}{w_i} = \frac{d_i}{\sqrt{2}}$ for $i < k$ and thus, for all $i \le j \le k$,

$$\begin{aligned} D_{ij} &= \sum_{\ell=0}^{j-i-1} d_{i+\ell} = \sqrt{2} \sum_{\ell=i}^{j-1} \frac{d_\ell}{\sqrt{2}} \\ &= \sqrt{2} \sum_{\ell=i}^{j-1} \log \frac{w_{\ell+1}}{w_\ell} = \sqrt{2} \log \frac{w_j}{w_i} \\ &= \sqrt{2\bigg(\log \frac{w_i}{g(w)} - \log \frac{w_j}{g(w)}\bigg)^2} \\ &= \sqrt{\sum_{\ell=1}^{k} \bigg(\log \frac{w_\ell}{g(w)} - \log \frac{w^{ij}_\ell}{g(w)}\bigg)^2} \\ &= d_{\mathrm{A}}(w, w^{ij}), \end{aligned}$$

where g denotes the geometric mean and d_{A} is the Aitchison distance, a metric on the unit simplex $\Delta^{k-1} \subseteq \mathbb{R}^k$ (6).

Let now $\psi \in S(K)$ be a fixed ground order of the elements of K and define the *compositional transformation* $\Psi_w \colon S(K) \to \Delta^{k-1}$ such that $\Psi_w(\pi) = P_\pi w$ where P_π is the unique permutation matrix with $P_\pi \psi = \pi$ (assuming vector notation of permutations). Intuitively, the vector $\Psi_w(\pi)$ assigns to every element $\{u, v\}$ of K in order of ψ a weight from w whose magnitude depends on the position at which $\{u, v\}$ appears in π. By assumption and design of w we obtain thus $d_{\mathrm{A}}(\Psi_w(\pi), \Psi_w(\pi^{ij})) = D_{ij}$, meaning that Ψ_w maps connection orders to compositional vectors (points on the simplex Δ^{k-1}) between which the Aitchison distance d_{A} locally predicts network dissimilarity.

The Aitchison geometry is isometric to regular Euclidean space as witnessed by the *isometric log-ratio transformation* $\mathrm{ilr} \colon \Delta^{k-1} \to \mathbb{R}^{k-1}$, a linear and isometric isomorphism (7). Thus, the *Euclidean transformation* $\Phi_w = \mathrm{ilr} \circ \Psi_w$ is a function that maps permutations onto points in Euclidean space and it can be computed efficiently. Its key property is that the distance between two output points agrees with the estimated dissimilarity between the two road networks that result from the input orders. This agreement is a close approximation for orders that differ in just two connections being exchanged, for larger changes it describes a smooth extrapolation from that estimate. Moreover, any point $x \in \mathbb{R}^{k-1}$ can be reversely associated with an order $\pi \in S(K)$ by computing the inverse transformation $\mathrm{ilr}^{-1}(x)$ and sorting the resulting vector.

Finally we show that the image of Φ_w is embedded in a hypersphere of radius $\|D\|_{\mathrm{F}}/(2\sqrt{k})$, where $\|\cdot\|_{\mathrm{F}}$ denotes the Frobenius norm of a matrix. Let $\pi \in S(K)$ arbitrary. Then, for the Aitchison norm $\|x\|_{\mathrm{A}} \coloneqq \sqrt{\langle x, x\rangle_{\mathrm{A}}}$ induced by the inner product $\langle x, y\rangle_{\mathrm{A}} \coloneqq \frac{1}{k} \sum_{i<j} \log \frac{x_i}{y_i} \log \frac{y_i}{x_i}$ on Δ^{k-1}, it is

$$\|\Phi_w(\pi)\| = \|\Psi_w(\pi)\|_{\mathrm{A}} = \|w\|_{\mathrm{A}} = \sqrt{\frac{1}{k} \sum_{i<j} \left(\log \frac{w_j}{w_i}\right)^2} = \frac{1}{\sqrt{2k}} \sqrt{\sum_{i<j} \left(\sum_{\ell=i}^{j-1} d_\ell\right)^2} = \frac{\|D\|_{\mathrm{F}}}{2\sqrt{k}}$$

where the first equality follows from $\Phi_w = \mathrm{ilr} \circ \Psi_w$ with ilr isometric while the second equality holds as the Aitchison metric is permutation-invariant and $\Psi_w(\pi) = P_\pi w$ with P_π a permutation matrix.

Continuous piecewise fit of network dissimilarity decay. We describe the procedure used to produce a functional fit $f \colon [1, k-1] \to \mathbb{R}_{>0}$ to a measurement vector $d_i \in \mathbb{R}^{k-1}_{\geq 0}$ such that $f(i) \approx d_i$ for all $i \in [k-1]$, where d_i denotes the average dissimilarity observed between a pair of sequential road networks of which one was produced from a randomly chosen connection order and the other from the same order but with the i-th and $(i+1)$-th entry exchanged. We assume prior knowledge that f is continuous, strictly decreasing, and interpolates between an initial exponential and a subsequent power law regime. We further make the simplifying assumption that the regime shift is abrupt and occurs at $h + \frac{1}{2}$ for some fixed $h \in \{2, \ldots, k-3\}$. Given that $d_i > 0$ for all $i \in [k-1]$, we define a small constant $\varepsilon > 0$ and solve the conic quadratic program

$$\begin{aligned}
\underset{\lambda, \gamma, \beta', \delta' \in \mathbb{R}}{\text{minimize}} \quad & \left\| \begin{bmatrix} 1 & 1 \\ \vdots & \vdots \\ h & 1 \end{bmatrix} \begin{pmatrix} \lambda \\ \beta' \end{pmatrix} - \begin{pmatrix} \log d_1 \\ \vdots \\ \log d_h \end{pmatrix} \right\| \\
& + \left\| \begin{bmatrix} \log(h+1) & 1 \\ \vdots & \vdots \\ \log(k-1) & 1 \end{bmatrix} \begin{pmatrix} \gamma \\ \delta' \end{pmatrix} - \begin{pmatrix} \log d_{h+1} \\ \vdots \\ \log d_{k-1} \end{pmatrix} \right\| \\
\text{subject to} \quad & \left(h + \frac{1}{2}\right) \lambda + \beta' = \log\left(h + \frac{1}{2}\right) \gamma + \delta', \\
& \beta' + \varepsilon \leq 0, \\
& \delta' + \varepsilon \leq 0
\end{aligned}$$

using standard software. From an optimum solution we recover the parameters $\beta \coloneqq e^{\beta'}$ and $\delta \coloneqq e^{\delta'}$ and define

$$f(t) \coloneqq \begin{cases} \beta e^{\lambda t}, & \text{if } t \in [1, h + \frac{1}{2}), \\ \delta t^{\gamma}, & \text{if } t \in [h + \frac{1}{2}, k-1]. \end{cases}$$

In practice, measurements $i \in [k-1]$ with $d_i = 0$ occur frequently; we process them by removing their associated rows (where $\log d_i$ would be undefined) from the vectors under the squared norms in the above problem. An alternative approach would be to replace such zeros with small values heuristically. Note further that the constant ε ensures that f is strictly decreasing even if data obtained from a poor sample size would suggest otherwise. An alternative approach would be to require only $\beta', \delta' \leq 0$ and treat solutions that violate $\beta', \delta' < 0$ as a failure to fit.

From the function f we finally obtain a strictly decreasing sequence $d' = (d'_i)_{i=1}^{k-1}$ via $d'_i \coloneqq f(i)$ that can be used instead of d to define the compositional weight vector w.

Extended results.

Extended analysis of the regimes of decay. We provide further empirical evidence for our hypothesis that, for intermediate values of the trade-off parameter α, the impact that small changes at increasing positions in a connection order have on the topology of the resulting network experiences a transition between two regimes: It is approximately described by an initial phase of exponential decay followed by a power-law decrease. More precisely, we extend the analysis presented in the main text to additional graph structures and designated site layouts (together referred to as "scenarios"). We assume $\alpha = \frac{1}{2}$ for all scenarios. The results are visualized in Figure S4 and are discussed below.

We analyze the following scenarios:

- Scenario A uses the same terrain graph as the experiment presented in the main text but a different random assignment for the $s = 20$ designated sites. Recall that the graph is obtained from a Delaunay triangulation of $n = 900$ points that are distributed uniformly at random in the unit square. All edge weights (base costs) are set to the Euclidean distance between the edge's endpoints.
- Scenario B uses the same terrain graph and number of designated sites as scenario A but the sites are chosen at random among a subset of vertices that are close to the corners of the unit square, leading to a clustered arrangement of the sites.
- Scenario C, on the other hand, concerns $s = 16$ well-distributed sites. To this end we place the sites at regular positions on a 46×46 square grid ($n = 2116$). Edge weights are uniform up to a small random perturbation that enforces uniqueness of least-cost paths with high probability.
- Scenario D considers a drawing of the complete graph on $n = s = 30$ vertices: Every pair of vertices shares an edge and every vertex is selected as a designated site. Edge weights are again set to the Euclidean distance between the connected sites. This represents the use of our model in a strongly non-planar setting and without possible junction nodes (non-site vertices).

We denote by $\delta(i)$ the mean network change that is induced by an exchange of the positions i and $i+1$ in a randomly selected connection order π of length $k = \binom{s}{2}$. Here, we do not fit a continuous function spanning both suspected regimes of δ (cf. section *Continuous piecewise fit of network dissimilarity decay* above and Figure S5 below). Instead, we fix the number of sites, s, as a possible breakpoint and we analyze the ranges $i \leq s$ and $i > s$ separately: To test whether $\delta(i)$ can be approximated by an exponential function in i for $i \leq s$, we perform linear regression in logarithmic space. (If $\log \delta(i)$ decays linearly in i, then $\delta(i)$ decays exponentially in i.) Likewise, to test whether $\delta(i)$ approximately follows a power-law for $i > s$, we perform linear regression in log-log-space. (If $\log \delta(i)$ decays linearly in $\log i$, then $\delta(i)$ is proportional to i raised to a negative power.)

For every scenario, we sample δ as follows: We first compute $N = 20\,000$ *base road networks* from randomly chosen *base connection orders*. Then, for every $i \in \{1, \ldots, k-1\}$, we exchange positions i and $i+1$ in each base order and compare the resulting sequential road networks with the corresponding base network (see section *Methods* of the main text for the measure used). The average over these N comparisons is then stored as the i-th entry of a vector of size $k-1$. Finally, we scale this vector such that its largest entry is 1 and we define $\delta(i)$ to be the i-th entry of the scaled vector. Note that it is possible for some $(i, i+1)$-exchange to induce no network change for any of the base orders. In this case, we cannot compute $\log \delta(i)$ and we ignore such a measurement. This can happen in particular for changes around later positions (when i is close to $k-1$) where the probability of affecting the resulting road network is low.

For all four scenarios tested, we find that the linear fit in log-space for $i \leq s$ is a good approximation for the transformed measurements $\log \delta(i)$, explaining 98% or more of the transformed data's variance ($r^2 \geq 0.98$). For scenarios A to C, a slight upward curvature of the measurements is visible, suggesting that the decay in this range is a bit slower than exponential. This is however consistent with our hypothesis of a transition towards a second regime of slower decay if this transition is smooth and begins before time s.

Also for the range of $s < i < k$, the linear fits in log-log-space adequately describe the transformed measurements. Here, our confidence is reduced by the fact that measurements disperse around the linear approximation for $i \gtrsim 2s$, leaving up to 34% of the transformed data's variance unexplained ($0.66 \leq r^2 \leq 0.74$). Except for scenario D, this variance is "well-behaved" in the sense that the regression line is rather accurate for $i < 2s$ despite a high sensitivity of the least-squares method towards outliers.

The results hint at two distinguishable regimes of decay, for which, respectively, an exponential function and a power function are adequate approximations. Our confidence in this regard is highest for $i \lesssim 2s$ as measurements for later positions are noisy at a resolution of 20 000 samples per position. As we test unordered, clustered and well-distributed sites as well as a non-planar graph structure, these findings appear to be structurally robust.

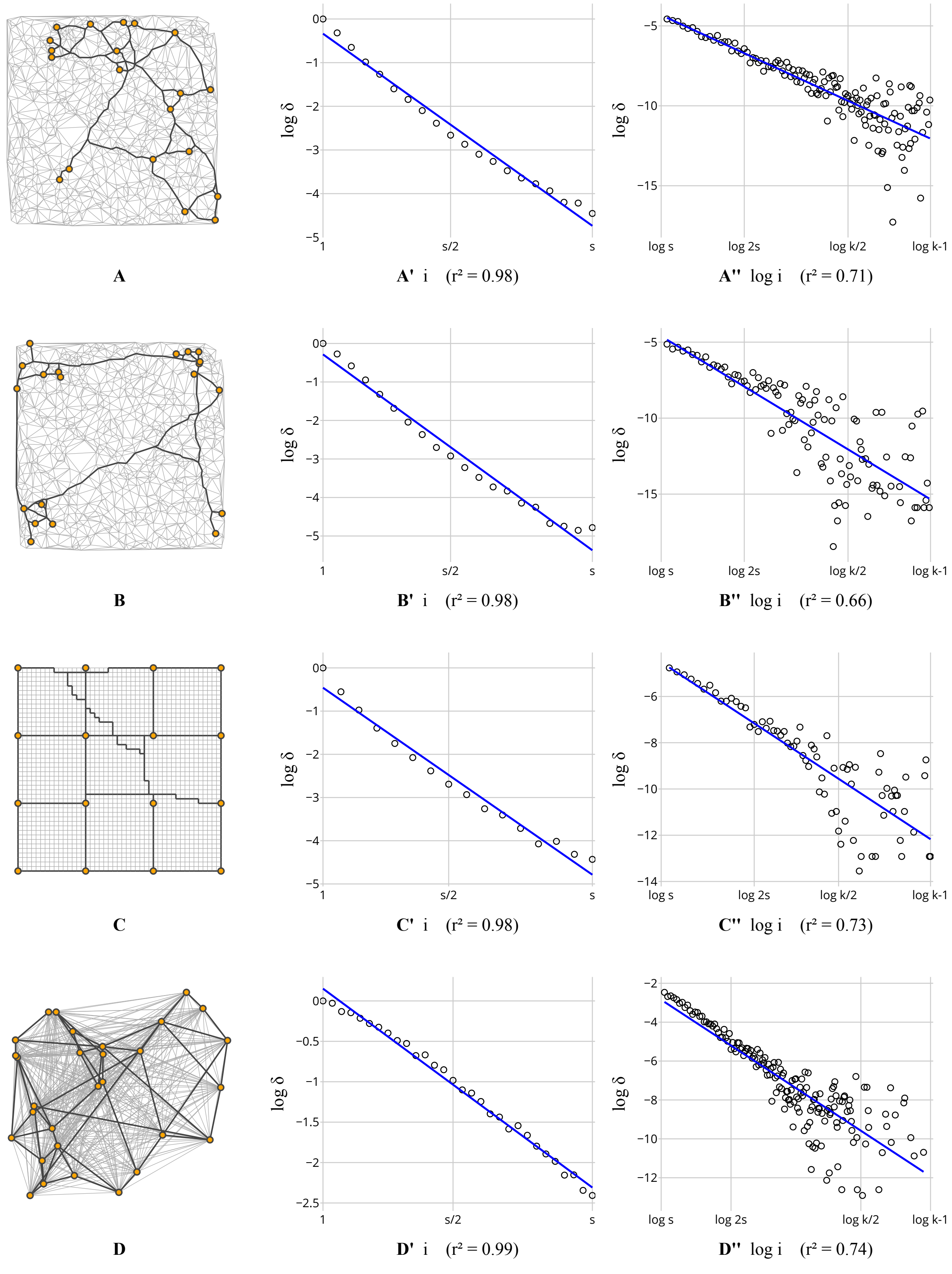

Fig. S4. Regimes of decay analysis for an idealized breakpoint at position s. Left panel: The tested scenarios A to D comprising a terrain graph (gray) and designated sites (orange), shown with an example road network (black, produced from a random connection order). Middle panel: The mean relative network change δ induced by an exchange of the i-th and $(i+1)$-th entry in a random connection order, for $i \leq s$ and drawn in logarithmic scale. The blue line describes a least-squares linear regression of the transformed measurements. An accurate fit would hint at an exponential relation between i and δ. Right panel: The same relation for the remaining indices $i > s$, drawn in log-log scale. An accurate fit of the regression line would hint at δ being proportional to a power of i. The value r^2 denotes the coefficient of determination of the regression line.

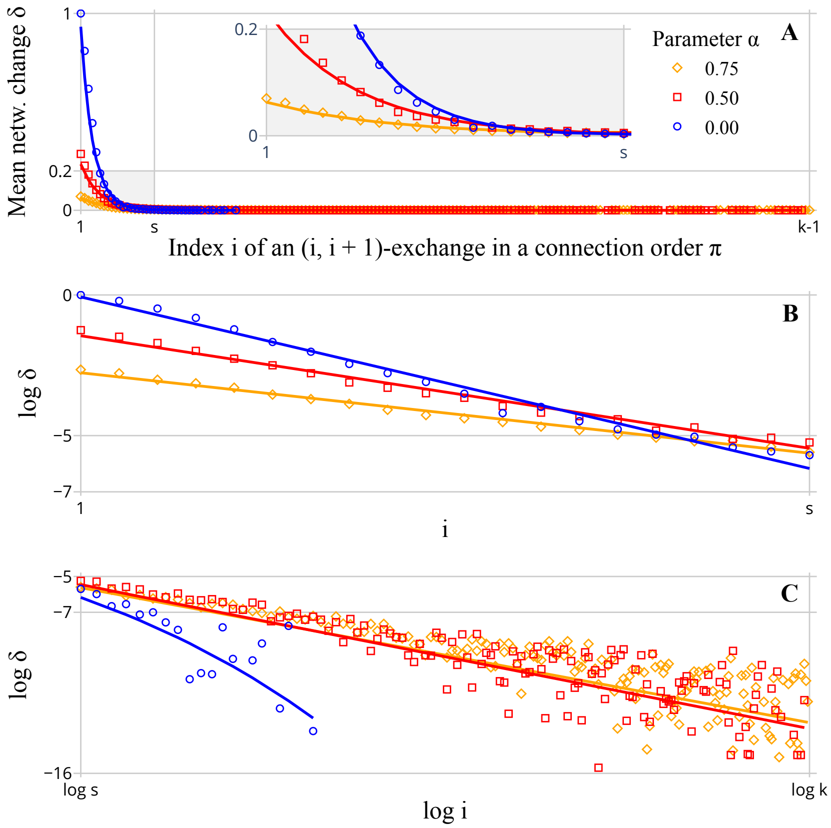

Fig. S5. Vanishing impact of atomic changes around later positions i in a connection order of length k. Extended version of a figure in the main text showing a linear (A) and a log-log plot (C) in addition to a logarithmic one (B). Unlike in Figure S4, the functional fits to the data are continuous (see section *Continuous piecewise fit of network dissimilarity decay* above for the procedure used).

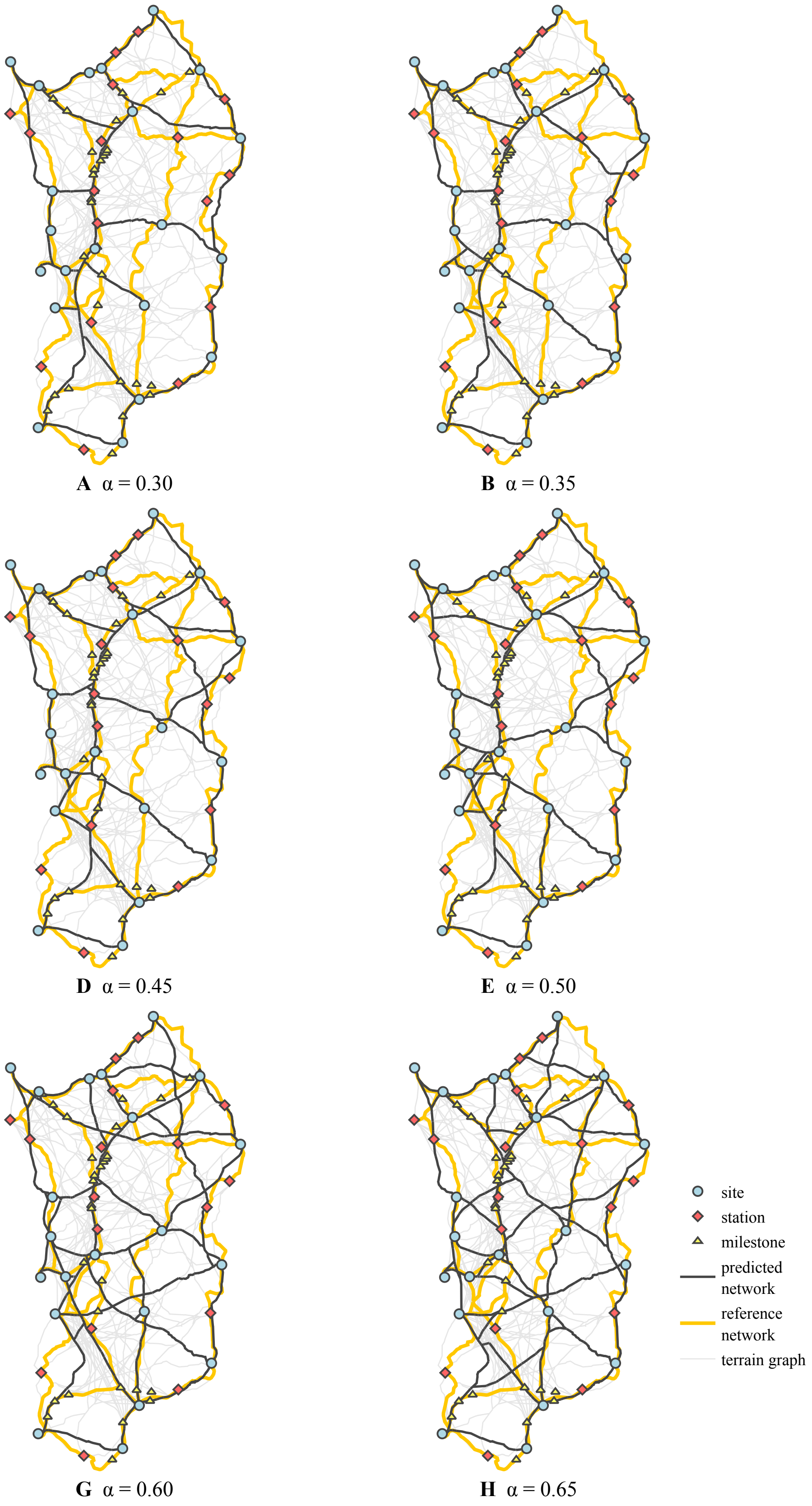

Fig. S6. Evidence-based reconstruction of the Roman road network on Sardinia. Extended version for $\alpha \in [0.3, 0.7]$. Each network is obtained from the first execution of the search heuristic described in the main text on the studied data set for the respective value of α.

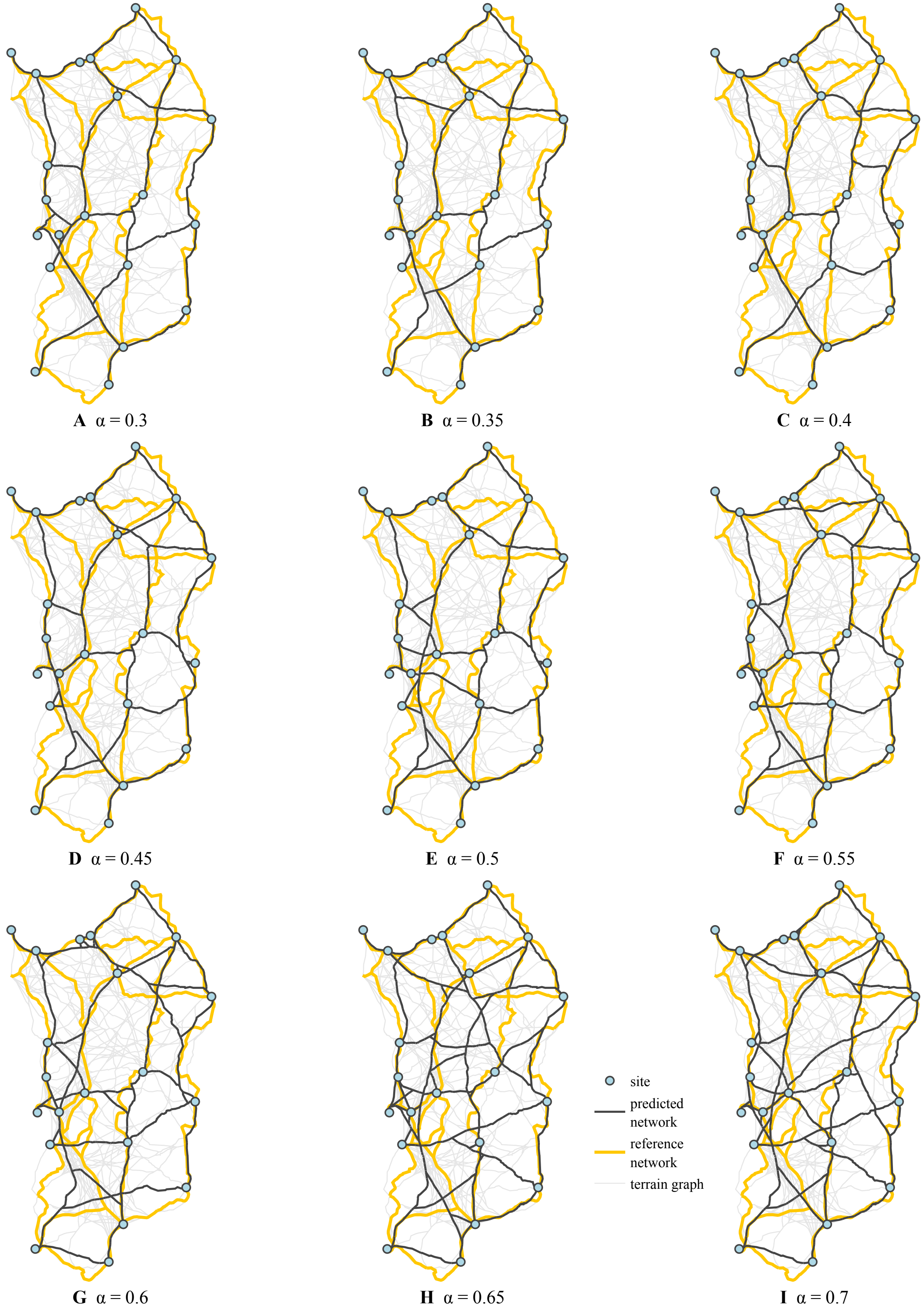

Fig. S7. Reconstruction of the Roman road network on Sardinia with the reference network available to the search procedure. The procedure minimizes the dissimilarity with the reference network as defined in the *Materials and Methods* section of the main text but with an increased Ramer-Douglas-Peucker tolerance of 5 km for improved performance. Each network is obtained from the first execution of the search heuristic given this objective and the respective value of α.

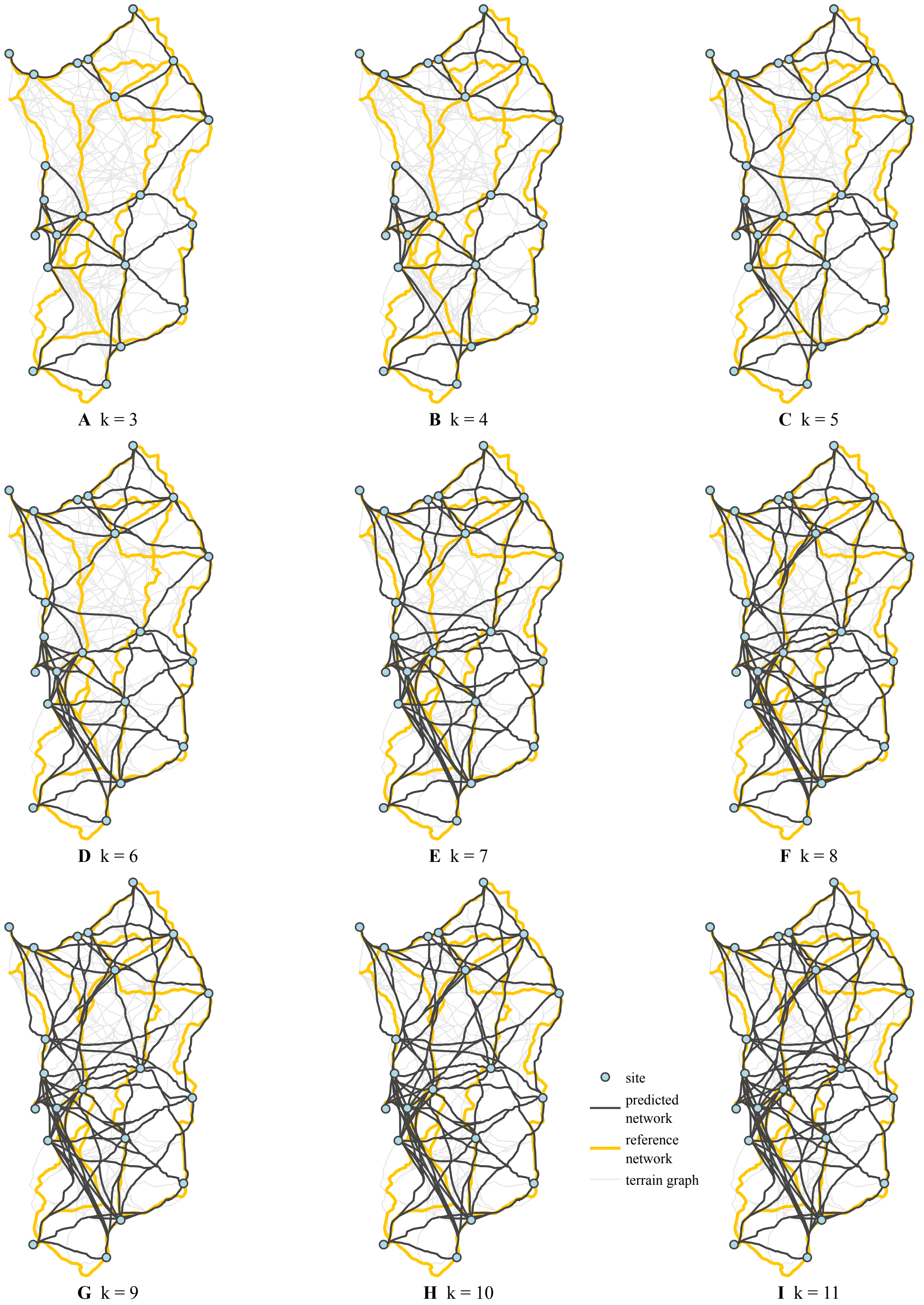

Fig. S8. Each site is connected by a least-cost path to its k cost-nearest neighbors. For $k < 3$ the network is not connected. At around $k = 6$ redundant roads start to appear that would not be cost-efficient to build or maintain in the real world.

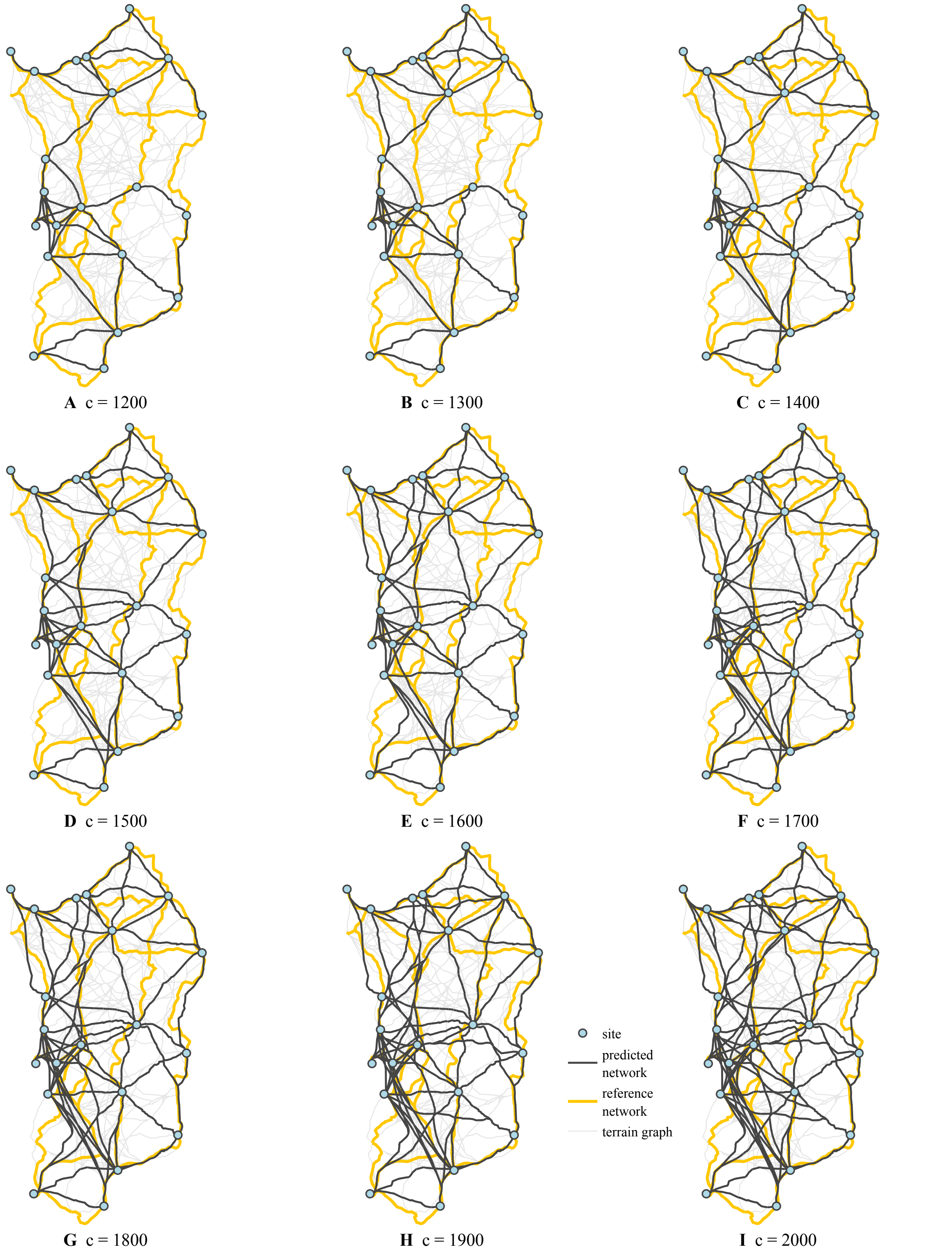

Fig. S9. Each site is connected by a least-cost path to all other sites at most c terrain cost units away. For $c \leq 1150$ the network is not connected. Redundant roads appear at around $c = 1500$.

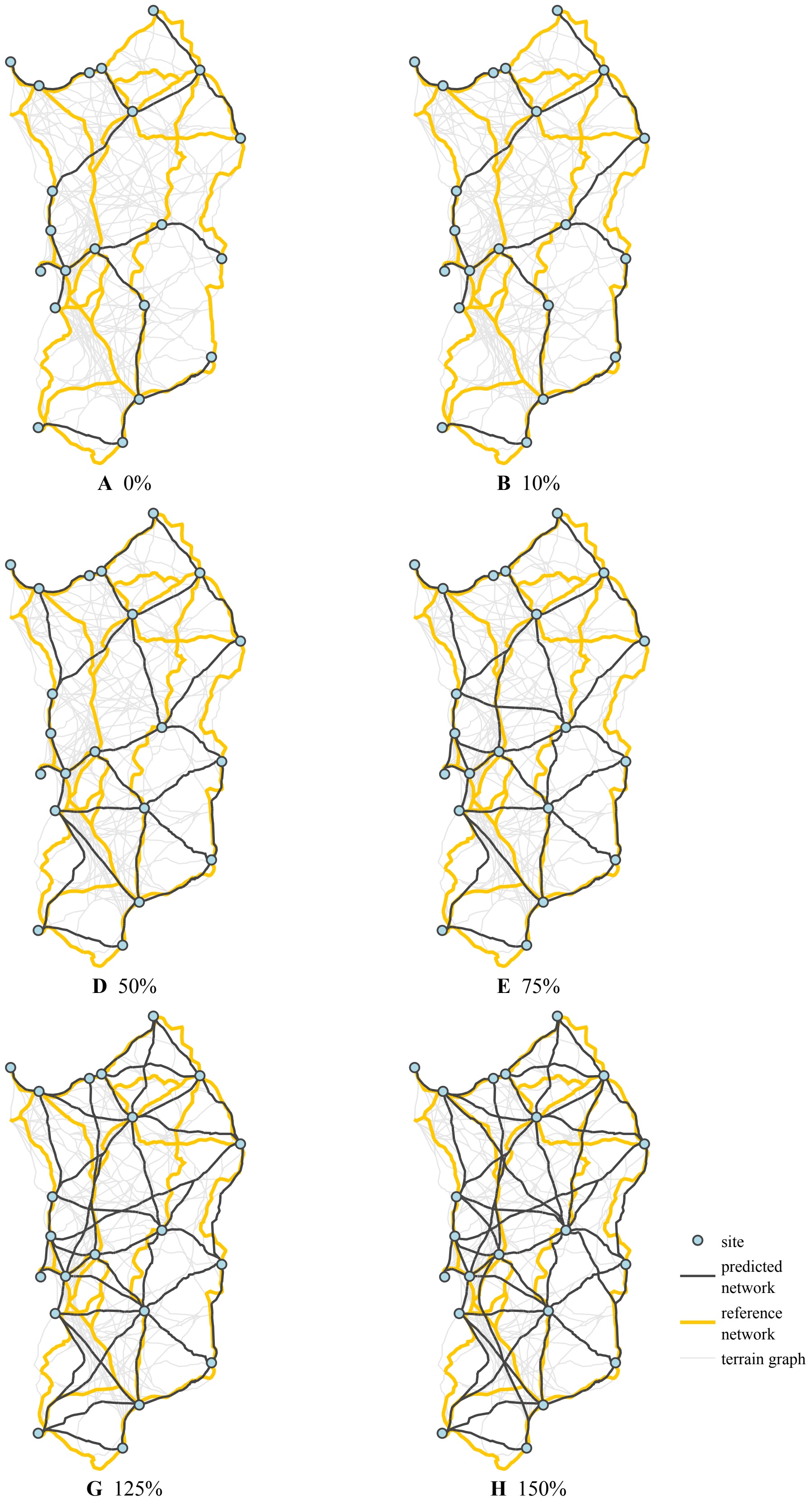

Fig. S10. Efficiency networks: Starting from a minimum-cost spanning tree (MST, see also Fig. S11*C*), successively add site-to-site paths that have the highest *efficiency*, that is the cost of the site-to-site path in the current network divided by the cost of the corresponding path in the terrain graph. Stop when the number of connections established like this is a certain percentage above that of the MST. This model is similar in spirit to the model proposed and could also be studied for varying connection orders. A key difference is that in our model, existing roads can be partially used. In an efficiency network, new roads are always added along the initial least-cost path between two sites.

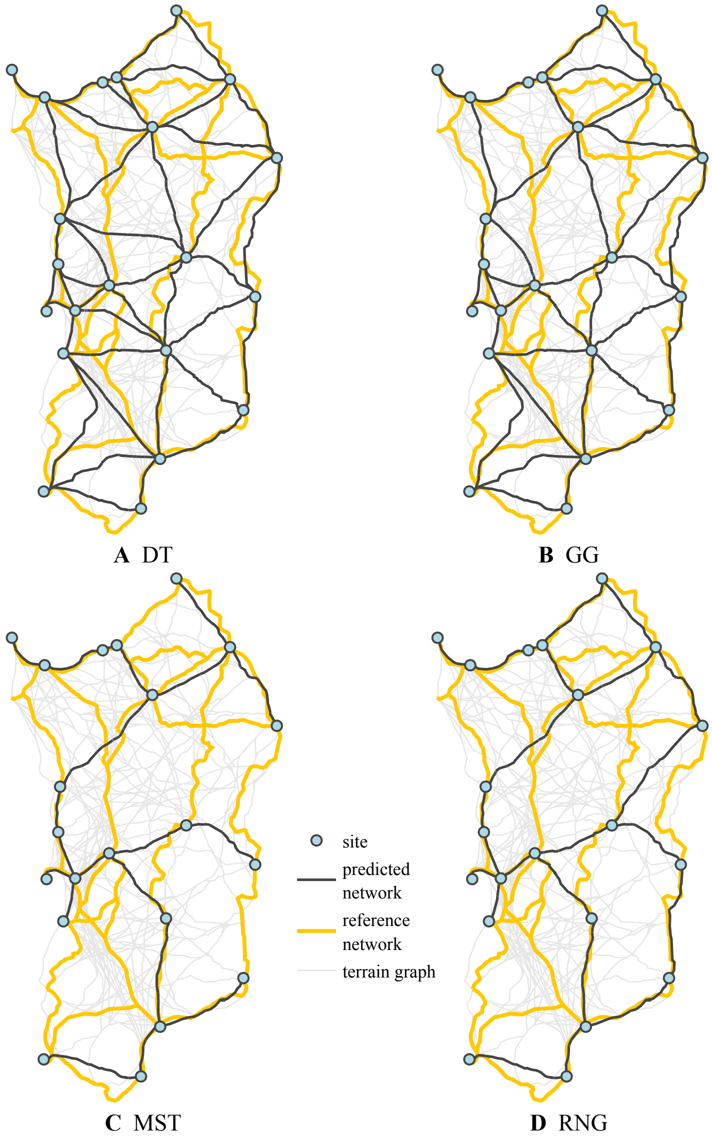

Fig. S11. Subgraphs of the generalized Delaunay triangulation with respect to the metric that is induced by the terrain graph. (A) Delaunay triangulation (DT): Connections are established along least-cost paths connecting the cells of a graph Voronoi diagram centered on the set of sites. There are no redundant roads and the reference network is matched reasonably well due to a guaranteed perimeter-circumscribing road that follows the coastline. However, the model cannot describe intersections or non-triangular faces that are prevalent in real-world road networks. Further, a circumscribing road might not be an adequate feature for inland use. (B) Gabriel graph: Site-to-site paths of the DT are kept if they do not intersect a Voronoi cell other than the two cells containing the path's endpoints. Omitting some of the chords of the DT, this model can describe non-triangular faces and does not have an unconditional circumscribing road but crossroads remain impossible, rendering it still unfit for the recovery of missing sites. (C) Minimum spanning tree: Connections are established along least-cost paths according to a minimum-weight spanning tree in the weighted complete graph whose vertices are the sites and whose edge weights are the corresponding site-to-site path costs. This baseline model produces networks that are too sparse to serve as meaningful network structure hypotheses. (D) Relative neighborhood graph: Two sites are connected if there is no third site that is closer to each of the two sites than the two are to each other. Unlike the MST this graph can contain cycles but it remains sparse in comparison with the reference network.

SI References

1. D. F. Manlove, "On the algorithmic complexity of twelve covering and independence parameters of graphs," *Discrete Applied Mathematics*, vol. 91, pp. 155–175, Jan. 1999.
2. M. Chlebik and J. Chlebikova, "Approximation hardness of dominating set problems in bounded degree graphs," *Information and Computation*, vol. 206, pp. 1264–1275, Nov. 2008.
3. D. Younger, "Minimum feedback arc sets for a directed graph," *IEEE Transactions on Circuit Theory*, vol. 10, pp. 238–245, June 1963.
4. C. Berge, *Graphs and Hypergraphs*, vol. 6. North-Holland Publishing Company, 1973.
5. P. Crescenzi, "A short guide to approximation preserving reductions," in *Proceedings of Computational Complexity. Twelfth Annual IEEE Conference*, IEEE, June 1997.
6. J. Aitchison, "The statistical analysis of compositional data," *Journal of the Royal Statistical Society. Series B (Methodological)*, vol. 44, no. 2, pp. 139–177, 1982.
7. J. J. Egozcue, V. Pawlowsky-Glahn, G. Mateu-Figueras, and C. Barceló-Vidal, "Isometric logratio transformations for compositional data analysis," *Mathematical Geology*, vol. 35, no. 3, pp. 279–300, 2003.
